# Bilaterian Giant Ankyrins Have a Common Evolutionary Origin and Play a Conserved Role in Patterning the Axon Initial Segment

**DOI:** 10.1371/journal.pgen.1006457

**Published:** 2016-12-02

**Authors:** Timothy Jegla, Michelle M. Nguyen, Chengye Feng, Daniel J. Goetschius, Esteban Luna, Damian B. van Rossum, Bishoy Kamel, Aditya Pisupati, Elliott S. Milner, Melissa M. Rolls

**Affiliations:** 1 Department of Biology, The Pennsylvania State University, University Park, Pennsylvania, United States of America; 2 Huck Institutes of the Life Sciences, The Pennsylvania State University, University Park, Pennsylvania, United States of America; 3 Department of Biochemistry and Molecular Biology, The Pennsylvania State University, University Park, Pennsylvania, United States of America; New York University, UNITED STATES

## Abstract

In vertebrate neurons, the axon initial segment (AIS) is specialized for action potential initiation. It is organized by a giant 480 Kd variant of ankyrin G (AnkG) that serves as an anchor for ion channels and is required for a plasma membrane diffusion barrier that excludes somatodendritic proteins from the axon. An unusually long exon required to encode this 480Kd variant is thought to have been inserted only recently during vertebrate evolution, so the giant ankyrin-based AIS scaffold has been viewed as a vertebrate adaptation for fast, precise signaling. We re-examined AIS evolution through phylogenomic analysis of ankyrins and by testing the role of ankyrins in proximal axon organization in a model multipolar *Drosophila* neuron (ddaE). We find giant isoforms of ankyrin in all major bilaterian phyla, and present evidence in favor of a single common origin for giant ankyrins and the corresponding long exon in a bilaterian ancestor. This finding raises the question of whether giant ankyrin isoforms play a conserved role in AIS organization throughout the Bilateria. We examined this possibility by looking for conserved ankyrin-dependent AIS features in *Drosophila* ddaE neurons via live imaging. We found that ddaE neurons have an axonal diffusion barrier proximal to the cell body that requires a giant isoform of the neuronal ankyrin Ank2. Furthermore, the potassium channel shal concentrates in the proximal axon in an Ank2-dependent manner. Our results indicate that the giant ankyrin-based cytoskeleton of the AIS may have evolved prior to the radiation of extant bilaterian lineages, much earlier than previously thought.

## Author Summary

The axon initial segment (AIS) is currently thought to be a distinguishing feature of vertebrate neurons that adapts them for rapid, precise signaling. It serves as a hub for the regulation of neuronal excitability as the site of action potential initiation and also acts as the boundary between the highly-specialized axon and the rest of the cell. Here we show that the giant ankyrins that structurally organize the AIS, and were thought to be vertebrate-specific, instead have an ancient origin in a bilaterian ancestor. We further show the presence of a giant ankyrin-dependent AIS-like plasma membrane boundary between the axon and soma in a *Drosophila* sensory neuron. These results suggest that the cytoskeletal backbone for the AIS is not unique to vertebrates, but instead may be an evolutionarily conserved feature of bilaterian neurons.

## Introduction

Polarized neurons with functionally distinct axons and dendrites are the foundation of the complex neuronal circuits in vertebrate nervous systems. The axon initial segment (AIS) plays a pivotal role in the function of polar vertebrate neurons as both a plasma membrane diffusion barrier that maintains the separate molecular identity of the axon, and as the site of action potential initiation. The presence of a plasma membrane diffusion barrier at the AIS that restricts lipid movement was first demonstrated in cultured neurons in 1992 [1]. Proteins were shown to have limited mobility at the AIS in 1999. In this latter study, the barrier was found to depend on actin: when actin was depolymerized, axonal and dendritic plasma membrane proteins leaked across the barrier into the other compartment [2]. Since then other elegant experiments on cultured neurons have confirmed the existence of the plasma membrane diffusion barrier [3], and have identified some of its molecular underpinnings.

Giant isoforms of the cytoskeletal crosslinker ankyrin G (AnkG) localize to the AIS and nodes of Ranvier in myelinated axons [4], and the longest 480 kD variant, formed by inclusion of an unusual 7.8 Kb exon [4], is required for AIS organization [5]. Increasing amounts of AnkG are associated with reduced diffusion [3], and targeting AnkG by RNAi allows increased diffusion of membrane tethered quantum dots in the AIS [6]. AnkG coordinates the AIS diffusion barrier in part by linking membrane proteins with the spectrin submembrane cytoskeletal network [7–9]. The high density of membrane proteins anchored at the AIS is believed to sterically limit the diffusion of other proteins and lipids through the plasma membrane [3, 8, 10]. Membrane proteins directly bound to AnkG are critical for AIS function as they include cell adhesion molecules and ion channels that control axon potential initiation. Thus AnkG is positioned as the central regulator of the AIS.

While ankyrins are present across the bilateria, AnkG itself is a vertebrate specific gene [11]. It is therefore unclear which, if any, aspects of the AIS exist outside vertebrates. AnkG is one of three vertebrate ankyrin paralogs (AnkB, AnkG and AnkR) that are believed to have arisen from a single ancestral ankyrin gene during two large scale duplications early in vertebrate evolution [5, 11]. Additional support for the idea that the AIS is a recent innovation comes from the observation that the AnkG binding sites on two vertebrate AIS-localized voltage-gated ion channels, voltage-gated Na^+^ channels and KCNQ K^+^ channels, can be traced back only as far as chordates and early vertebrates, respectively [12]. The AIS is thus frequently cited as a key evolutionary innovation within the vertebrate lineage that, together with myelin, allows the rapid, precise signaling necessary for our complex nervous systems [5, 11–13].

Bilaterian ankyrins share a common motif structure that includes an N-terminal domain of 24 ankyrin repeats involved in transmembrane protein tethering, a central core involved in spectrin binding that consists of a tandem array of two ZU5 domains and one UPA domain, followed by a death domain (DD) of uncertain function. The ankyrin gene family can be traced as far back as cnidarians, a sister group to the bilaterians, although it has been suggested that this complete domain structure is absent in cnidarians and thus is bilaterian-specific [11]. The evolutionary history of the long exon present in vertebrate AnkG, which has been shown to be critical to AIS function [5], is less clear. The current view is that the long exon was inserted into a vertebrate AnkG/AnkB ancestor only after the first of the two genome-scale rounds of duplications in vertebrates [5, 11, 14]. It is not present in vertebrate AnkR, which was a direct product of the first vertebrate genome-scale duplication, and has been reported as absent from ankyrin in the tunicate *Ciona intestinalis* [5]. Tunicates separated from the vertebrate lineage early in chordate evolution prior to the vertebrate ankyrin gene duplications. Interestingly, the ankyrin binding domain present on chordate voltage-gated Na^+^ channels predates these vertebrate gene duplications [12], suggesting that ankyrins may have already been playing a role in the clustering of at least some axonal ion channels prior to the emergence of vertebrate AnkB and AnkG.

Giant neuronal isoforms including unusually long exons have been found in the *Drosophila* neuronal ankyrin, *Ank2* [15, 16], and the lone *C*. *elegans* ankyrin (*unc-44*) [17, 18], but it has been postulated that these long exons have a separate evolutionary origin from the vertebrate long exon because 1) they do not share significant sequence homology with the long exon of vertebrate AnkG, 2) they are inserted downstream rather than upstream of the DD, and 3) they have not been found in intervening species such as *Ciona* [14]. The giant isoform of *C*. *elegans unc-44* is required for normal axonal growth [19], and an unidentified isoform of *unc-44* is also required to maintain axon identity and concentrate the microtubule organizer CRMP to the proximal axon [20]. *Drosophila Ank2* contains two long exons, XL and L, and the giant isoforms they encode work together to regulate axon diameter and synapse stability through microtubules [15, 16, 21]. Interestingly, genetic deletion of *Ank2* leads to ectopic branching in the proximal axon of *Drosophila* sensory neurons [22]. However, a possible role for these long invertebrate ankyrins in generating AIS-like structures has not been examined to date.

A late vertebrate origin for the AIS would contrast with findings that other fundamental features of polar neurons evolved much earlier, at least prior to the divergence of protostomes and deuterostomes, the two major bilaterian lineages. Many families of neuronal ion channels and receptors have origins in basal metazoans or even protozoans [23–29], and diversification of the major neuronal voltage-gated ion channels was complete prior to the divergence of bilaterians and cnidarians [26, 30–34]. Polar neurons with distinct axons and dendrites can be found in protostome invertebrate model organisms such as *Drosophila* and *C*. *elegans* [35]. Axons and dendrites in vertebrates, *Drosophila* and *C*. *elegans* can all be distinguished by shared differences in microtubule polarity: axons contain almost exclusively plus-end-out microtubules, while dendrites contain high percentages of minus end-out microtubules [35–39]. In addition to axon-dendrite polarity, γ-neurons in the *Drosophila* mushroom bodies have patterned concentrations of ankyrin and K^+^ channels in a proximal domain reminiscent of the vertebate AIS [40]. We therefore reasoned that at least some structural features of the vertebrate AIS may predate the divergence of the protostome and deuterostome lineages. In particular, the maintenance of distinct axons and dendrites in invertebrates suggests a possible role for an ankyrin-based diffusion barrier. Although it is not known whether invertebrate neurons have an AIS-like diffusion barrier, they do at least have the capacity to establish plasma membrane diffusion barriers. It was recently shown that *Drosophila* motor neurons in culture generate a diffusion barrier half way out their nascent axon as they develop [41]. The relationship between this barrier and the mammalian AIS diffusion barrier or its dependence on ankyrins is not known.

We re-examined the possible evolutionary origins of the ankyrin-based AIS diffusion barrier taking a two-pronged approach. Here we report a broad evolutionary analysis of ankyrins, including giant isoforms encoded by long exons, and a functional analysis of the proximal axon of a model *Drosophila* sensory neuron. We find substantial evidence for a common evolutionary origin for giant ankyrins in a bilaterian ancestor. Furthermore, we present evidence for an AIS-like diffusional barrier *in vivo* in the proximal axon of a *Drosophila* sensory neuron, and patterns of ion channel localization, that depend on giant Ank2 isoforms. These results suggest that at least some of the neuronal functions of giant ankyrins evolved much earlier than previously thought, before the divergence of the major bilaterian lineages.

## Results

### Complete ankyrin domain structure is represented in cnidarians and bilaterians

**Fig 1:**
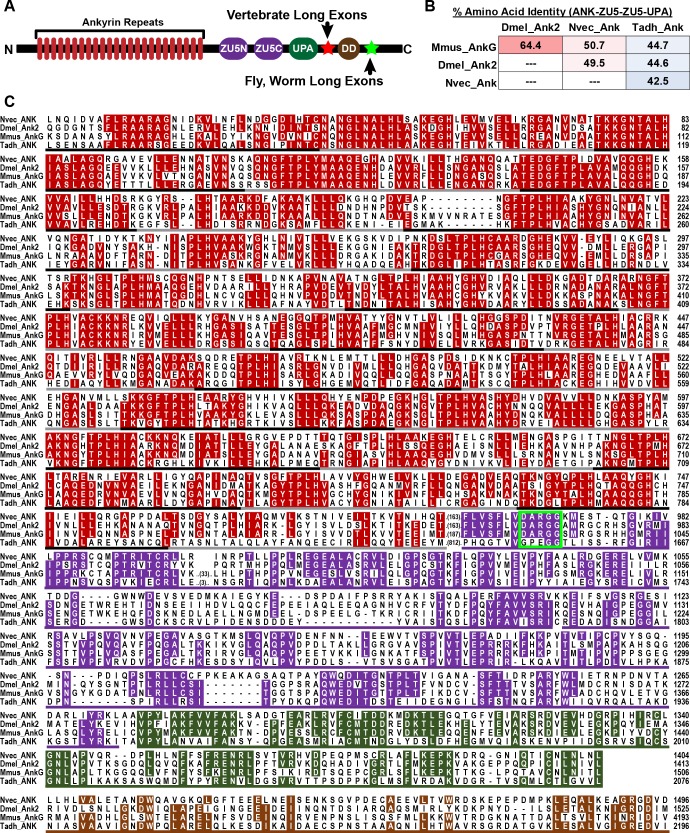
Ankyrins are highly conserved between bilaterians, cnidarians and placozoans. (A) schematic diagram of a canonical ankyrin protein showing the 24 ankyrin repeats, the ZU5-ZU5-UPA cassette and the death domain (DD). The position of the long exon included in giant isoforms is shown for vertebrates (red star) and protostome invertebrates (green star). (B) Pairwise amino acid identity shared between mouse AnkG (Mmus_AnkG; Bilateria, deuterostome), *Drosophila melanogaster* Ank2 (Dmel_Ank2; Bilateria, protostome), *Nematostella vectensis* Ank (Nvec_Ank; Cnidaria) and *Trichoplax adhaerens* Ank (Tadh_Ank; Placozoa) for the shown alignment. (C) Amino acid alignment of core conserved domains is shown for bilaterian, cnidarian and placozoan ankyrins. Gene name is given at the left margin and amino acid position at the right margin. Residues identical in at least 3/4 sequences are shaded according to the domain color codes shown in (A). Domains boundaries are indicated with underlines; the 24 ankyrin repeats are underlined with alternating black and gray lines for clarity. The spectrin binding motif of vertebrate ankyrins is highlighted with a bright green outline.

We first explored the evolutionary origin of ankyrins using TBLASTN searches of available sequence data from early metazoan lineages (cnidarians, placozoans, sponges, ctenophores) and choanoflagellates, the most closely related protozoans to the metazoan lineage. S1 Table lists species and data type searched (genome or transcriptome), and a summary of ankyrin genes found, and amino acid sequences of the ankyrins are provided in S2 Table. We found one complete ankyrin with 24 ankyrin repeats, the ZU5-ZU5-UPA cassette and the DD in each of four cnidarian species (*Nematostella vectensis*, starlet sea anemone [26]; *Orbicella faveolata*, star coral; *Acropora millepora*, stony coral [42]; and *Exaiptasia pallida*, brown anemone [43]), and in the placozoan *Trichoplax adherens* [44]. Fig 1 shows an amino acid alignment of the core domains between mouse AnkG, *Drosophila* Ank2 and the ankyrin orthologs from a cnidarian (*Nematostella vectensis*, starlet sea anemone) and a placozoan (*Trichoplax adhaerens*). 336/1205 positions (28%) in the alignment are identical in all 4 sequences and a further 345 positions (29%) are identical in 3 of 4 sequences. *Drosophila* Ank2 and mouse AnkG share 64.4% amino acid identity across the Ank-UPA core, while *Nematostella* shares ~50% identity to the bilaterian ankyrins. Lower sequence identity for *Nematostella* ankyrin is expected given that cnidarians and bilaterians diverged well before the radiation of bilaterians; a similar small drop in sequence identity has been consistently observed for cnidarian ion channels compared to their bilaterian orthologs despite high conservation of functional properties [30–33, 45–47]. Similarly, *Trichoplax* ankyrin, shares only 42.5–44.7% identity with the other sequences, consistent with the hypothesis that placozoans are the earliest diverging member of the eumetazoan clade, which includes placozoans cnidarians and bilaterians [27, 44, 48].

In contrast to our findings in cnidarians and placozoans, we were unable to identify ankyrin genes that contained ankyrin repeats with the ZU5-ZU5-UPA cassette in nine sponge species (one genome and eight transcriptome assemblies, [28, 49]), six ctenophores species (2 genomes and 6 transcriptome assemblies, [25, 27]) or two choanoflagelletes (2 genome assemblies, [29, 50]) (S1 Table). Thus canonical ankyrins may have evolved in a common ancestor of eumetazoans after separation from the sponge and ctenophore lineages. This finding contrasts with a previous report that canonical ankyrins are bilaterian-specific and cnidarian ankyrins contained only the 24 ankyrin repeats and the first ZU5 domain [11]. However, it is consistent with the observation that many neuronal signaling proteins such as voltage-gated ion channel gene families also diversified at this time [24, 26, 31, 44].

### Giant ankyrins are found only bilaterians

As giant ankyrins are important for ankyrin function at the AIS, we examined ankyrin loci and gene predictions from invertebrate bilaterians, cnidarians and placozoans for evidence of giant isoforms. Vertebrate giant ankyrins are generated by inclusion of a long exon after the UPA domain. We therefore searched for potential long exons (defined here as > 3 Kb) anywhere downstream of the UPA domain in invertebrate genomes and corresponding giant isoforms in transcriptome databases. Cnidarian and placozoan ankyrins do not appear to have giant isoforms encoded by long exons based on two lines of evidence. First, we found no giant isoforms in transcriptome data from three cnidarian species: *Nematostella vectensis*, *Orbicella faveolata* and *Acropora millepora*. Second, there were no ORFs > 1 Kb in genome sequence between the UPA domain exons of the ankyrin gene and the adjacent downstream gene in genome drafts for the cnidarians *Nematostella vectensis*, *Orbicella faveolata* and *Exaptasia pallida* and the placozoan *Trichoplax adhaerens*. These results suggest that while canonical ankyrins were present in a eumetazoan ancestor of cnidarians, placozoans and bilaterians, giant ankyrins are likely to be bilaterian-specific.

### Giant ankyrins including long exons are present throughout the bilateria

**Fig 2:**
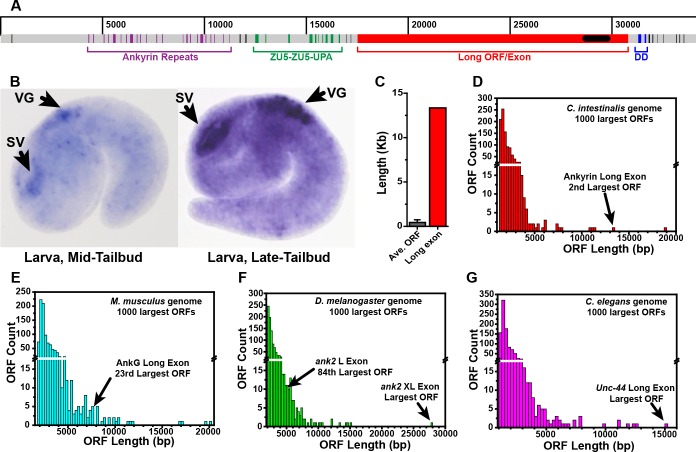
*Ciona intestinalis* ankyrin has a long exon comparable to mouse, fly and worm that can encode giant isoform(s). (A) A map of the Ciona intestinalis ankyrin locus showing the position of exons (color coded by domain) comprising an ankyrin ortholog gene prediction relative to the position of a giant ORF (red) that could represent a long exon. (B) *In situ* hybridization of mid (left) and late (right) tail bud stage larvae with a probe corresponding to an EST cluster from within the potential long exon (black box in A) shows expression in the sensory vesicle (SV) and the visceral ganglion (VG), which together comprise the bulk of the larval nervous system. Pictures are publically available from the Aniseed database (**A**scidian **N**etwork for **I**n **S**itu **E**xpression and **E**mbryological **D**ata; http://www.aniseed.cnrs.fr/) where additional pictures and methods can be found (gene prediction KH.C1.943). (C) Comparison of the Ciona ankyrin long exon size with that of the average size of all ORFs (defined as > 300 nucleotides without a stop codon) in the Ciona genome draft. (D) Length distribution for the largest 1,000 ORFs in the Ciona genome in bins of 250 bp, reveals that the Ciona ankyrin long exons sits in the 2^nd^ largest ORF in the genome. (E-G) Similar length distribution graphs for the largest 1,000 ORFs in mouse, *Drosophila melanogaster* and *C*. *elegans* with the rank of long ankyring exons indicated.

To understand when in bilaterian evolution giant ankyrins appeared, and to determine whether insertion of long exons was likely to have occurred as a single event or multiple times, we analyzed ankyrin genes from key bilaterian species. We began with the lone ankyrin gene from the tunicate *Ciona intestinalis* because the absence of a gene prediction for a giant ankyrin with a long exon has been cited as evidence that giant ankyrins appeared only after the genome-wide duplications in vertebrates [5, 14]. We mapped a predicted *Ciona intestinalis* ankyrin ortholog (XM_009864015) containing the complete Ankyrin-ZU5-ZU5-UPA-DD structure (but no long exon) to the *Ciona intestinalis* genome draft. The gene prediction comprises 48 exons spanning ~33 Kb, with ZU5-ZU5-UPA cassette encoded in exons 27–36 and the DD encoded in exons 39 and 40 (Fig 2A). Interestingly, we found a 14 Kb gap separating exons 38 and 39 which contained an unbroken 13.4 Kb ORF. A small EST cluster (clones cien56932, cijv010l23, cijv033b07, cidg851d03, cilv031p13 and 031ZG09) mapping within this ORF is expressed in the nervous system (Fig 2B, [51]), indicating that the ORF could encode a long exon for a giant ankyrin isoform. We therefore used PCR on cDNA from whole larvae to verify that this long ORF contains exonic sequence contiguous with the UPA domain on the 5’ end and the DD on the 3’ end; The intron/exon junctions we verified by PCR suggest a 13,344 bp exon lies within the long ORF. The giant ankyrin isoform predicted for inclusion of this exon is provided in S2 Table.

To determine whether insertion of such a long ORF is a frequent event, we analyzed ORF length in the *Ciona* genome draft. We found 81,915 ORFs > 300 bp in the *Ciona* genome assembly with an average size of 436 ± 308 (S.D.) bp. The predicted long exon in the ankyrin gene is ~42 standard deviations larger than this average and is the 2^nd^ largest ORF present in the entire genome assembly (Fig 2C and 2D, S3 Table). Similar analyses show that the mouse AnkG long exon is in the 23 longest ORF in the mouse genome; the *Drosophila Ank2* XL and L exons correspond to the largest and 84^th^ largest ORFs in the *Drosophila* genome, and the *C*. *elegans* ankyrin (*unc-44*) long exon sits within the longest ORF in the *C*. *elegans* genome (Fig 2E–2G, S3 Table). Because the long ORFs in *C*. *intestinalis*, *Drosophila*, *C*. *elegans* and mouse ankyrin genes are all at the upper end of ORF size in their respective genomes, we considered that such uncommonly large ORFs might not be inserted as independent evolutionary events. Based on this initial finding of an unusually large ORF in the *C*. *intestinalis* ankyrin, we analyzed ankyrin genes in representative species across the Bilateria.

We found evidence for giant ankyrins in genome and/or transcriptome data in all phylogenetic groups of bilaterians we examined including tunicates, cephalochordates, echinoderms, mollusks, annelids and diverse arthropods. Amino acid sequences and accession numbers of these ankyrins are provided in S2 Table, and long exon lengths and position (relative to the DD) are provided in S4 Table. 23/36 bilaterian ankyrins we examined have long exons and can encode giant isoforms, and an additional ankyrin (from the giant earthworm *Glossocolex paulistus*) has a giant isoform in transcriptome data. 8 bilaterian ankyrin loci, including 5 vertebrate AnkR orthologs and two insect *Ank* orthologs, do not have a potential long exon in genome sequence and thus are not able to encode giant isoforms. We identified four additional short ankyrin isoforms exclusively in transcriptome data from tunicates, echinoderms and *Glossoscolex*. Because we had no corresponding genome data for these species, we were not able to determine whether these genes also encode alternate giant isoforms.

While most ankyrin loci contained a single long exon, 8/23 of the ones we examined had two tandem long exons, including *Drosophila* (S4 Table). Two long exons in ankyrin from the cephalochordate *Branchiostoma floridae* are homologous and thus likely the result of a recent duplication of a single ancestral exon (S1A Fig). Multiple long exons in the AnkB ortholog in zebrafish, green sea urchin ankyrin (*Lytechnicus variegatus*) and two horshoe crab (*Limulus polyphemus*) ankryins appear to be the result of recent intron insertions that split a single ancestral exon (S1B Fig). The origin of the two long exons in *Drosophila Ank2*, its flour beetle (*Tribolium castaneum*) ortholog and *Aplysia* ankyrin are less clear because the exons are not homologous and there are no ancestral homology blocks split by an intron.

The size of the long ankyrin exons was quite variable, with a mean of 8103 ± 4850 bp (S.D., n = 30). The bulk of the *Drosophila* XL exon has a core array of 93 highly-conserved repeats encoding 76 amino acids which has been postulated to serve as a molecular spacer, although only a fraction of the array is required for *Ank2* function in maintaining axon diameter [21]. Arrays of homologous repeats are not present in vertebrate AnkGs [14], and we found repeat arrays in only a subset of the long exons (S4 Table). Repeat numbers varied from 5 to > 160, the size of repeats varied from 36 bp– 228 bp, and only repeats from very closely related species had recognizable homology. Thus repeats are not a feature of ankyrin long exons that is conserved over large evolutionary distances. We conclude that giant ankyrins that include sequences encoded by very long exons/ORFs are present in all major bilaterian groups. While the ORFs are always thousands of nucleotides in length, their size and repeat content varies considerably.

### Giant ankyrins are ancestral in bilaterians

**Fig 3:**
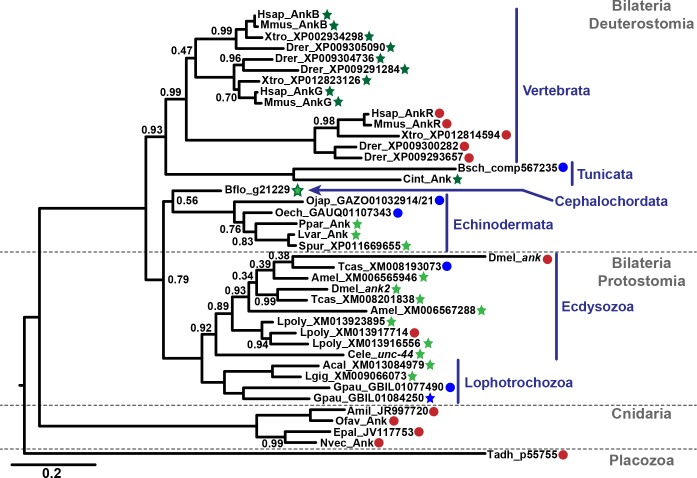
Maximum Likelihood phylogeny of the ankyrin gene family. The tree is midpoint rooted for display, major phylogenetic groups are indicating in grey text and separated with dashed lines for largest groups and blue lines and text for subgroups (labels at right), and the scale bar indicates sequence distance. Protein names are given at branch tips and node values indicate frequency of node recovery in 100 bootstrap replications (not shown if 1). Dark green and light green stars indicate genes that encode giant ankyrins with a long exon upstream or downstream of the death domain (DD), respectively. We found long exons but no DD for Branchiostoma ankyrin (Cephalochordata, light green star/dark green outline). Red circles indicate ankyrin genes with no long exons that are incapable of encoding giant isoforms. Blue circles indicate short and giant isoforms for which only transcriptome data was available; we could not therefore determine whether these genes are also capable of encoding giant isoforms. The blue star indicates a transcriptome-only giant isoform for which we were not able to verify the presence of a long exon. Aligned sequences on which the phylogeny is based and full sequences for each protein are included in S2 Table. Species prefixes in protein names are as follows: Acal, *Aplysia californica* (sea hare); Amel, *Apis mellifera* (honey bee); Amil, *Acropora millepora* (stony coral); Bflo, *Branchiostoma floridiae* (florida lancelet); Bsch, *Botryllus schlosseri* (golden star tunicate); Cele, *Caenorhabditis elegans* (nematode); Cint, *Ciona intestinalis* (vase tunicate); Dmel, *Drosophila melanogaster* (fruit fly); Drer, *Danio rerio* (zebrafish); Epal, *Exaiptasia pallida* (brown sea anemone); Gpau, *Glossoscolex paulistis* (giant earthworm); Hsap, *Homo sapiens* (human); Lgig, *Lottia gigantea* (owl limpet); Lpoly, *Limulus Polyphemus* (horseshoe crab); Lvar, *Lytechnicus variegatus* (green sea urchin); Mmus, *Mus musculus* (mouse); Nvec, *Nematostella vectensis* (starlet sea anemone); Oech, *Ophiocoma echinata* (brittle sea star); Ofav, *Orbicella faveolata* (star coral); Ojap, *Oxycomanthus japonicus* (feather star); Ppar, *Parastichopus parvimensis* (sea cucumber); Spur, *Stronglyocentrotus purpuratus* (purple sea urchin); Tcas, *tribolium castaneum* (red flour beetle), and Xtro, *Xenopus tropicalis* (clawed frog).

To better understand how the current set of ankyrins evolved in bilaterians including the mammalian short AnkR, giant AnkG, AnkB, and the protostome giant ankyrins, we built a maximum likelihood phylogeny for the ankyrin family using the ankyrin repeats and the ZU5-ZU5-UPA cassette (Fig 3). If giant ankyrins evolved independently in vertebrates and protostomes (like *Drosophila*) as has been previously proposed [5, 14], then we would expect the ankyrin genes without long exons to connect to the base of the bilaterian ankyrin tree through a continuous series of nodes, and giant ankyrins to arise multiple times from separate nodes. Instead, it is the short ankyrins that arise from isolated nodes in vertebrates (AnkR), insects (*Drosophila Ank*) and *Limulus* (Fig 3, red circles). In contrast, the giant ankyrins, which we found in all bilaterian phyla examined, all connect to the base of the bilaterian ankyrin branch through nodes that connect to other giant ankyrins (Fig 3, green stars). The most parsimonious interpretation of the phylogeny is that ankyrins acquired a long exon and the ability to encode giant isoforms prior to the radiation of extant bilaterian phyla. For example, multiple genomes reveal that echinoderms, cephalochordates and tunicates have a single ankyrin gene with at least one long exon, implying that the long exon is ancestral in deuterostomes. Even if we assume that short ankyrins we found exclusively in transcriptome data (from echinoderms, tunicates, and annelids, Fig 3, blue circles) are encoded by ankyrin genes that lack long exons, the giant ankyrins remain connected across the phylogeny (Fig 3).

**Fig 4:**
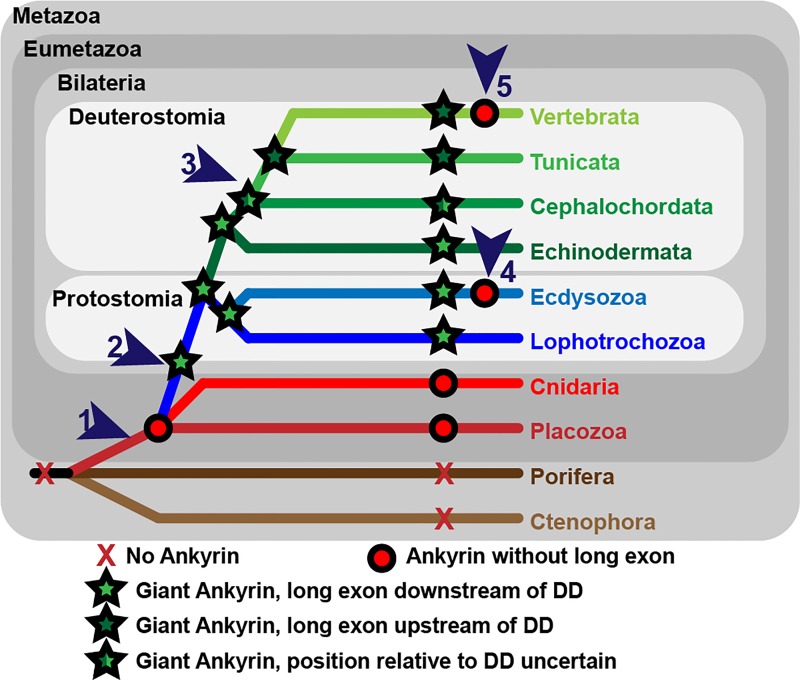
Model for ankyrin family evolution based on the phylogenetic distribution of short and giant ankyrins. **Numbered arrowhead indicate key points in Ankyrin evolution as follows:** (1) Ankyrins likely first evolved in an ancestor of extant eumetazoans because they can be found in placozoans, cnidarians and bilaterians, but are absent from sponges (Porifera) and comb jellies (Ctenophora). The ancestral Ankyrin lacked the long exon and could not encode giant isoforms. (2) The long exon was most likely inserted after divergence of the eumetazoans in an ancestor of extant bilaterians, because giant ankyrins encoded by inclusion of a long exon are present in all major groups of bilaterians that we examined. The original long exon was presumably inserted downstream of the death domain (DD) because it is found in this position in both protostomes and early diverging deuterostome lineages. (3) The long exon switches position prior to the emergence of chordates, which carry the long exon upstream of the DD. The position in cephalochordates is uncertain as we were unable to identify a DD in *Branchiostoma*. The DD has low homology and is typically split over several exons and is thus difficult to identify if it is not present in automated gene prediction and transcriptome data is not available. The ancestral long exon has been lost multiple times in bilaterian evolution including (4) two separate lineages within the Ecdysozoa and (5) the vertebrate AnkR lineage.

As phylogenies based on single genes do not always recapitulate species phylogenies based on more comprehensive data, we mapped the information about presence of giant ankyrins onto the known evolutionary relationships of major animal groups (Fig 4). Based on the types of ankyrins present in the extant species, we made predictions about whether giant ankyrins existed at each evolutionary node (Fig 4). Using this summary, several arguments against a single origin for giant ankyrins can be addressed.

One argument against a common, ancestral origin for giant ankyrins is that the position of the long exon relative to the DD is different in *Drosophila* and mammals [14]. In Figs 3 and 4 the two positions are shown as light green and dark green stars, and mixed stars indicate a single cephalochordate sequence where the DD could not be identified. Interestingly the *Drosophila* position, after DD, is found quite broadly throughout protostomes (Fig 4). Most significantly this position after the DD is also found in multiple echinoderm ankyrins. Echinoderms are deuterostomes and this suggests that the position of the long exon after DD position is ancestral in both protostomes and deuterostomes (Fig 4). The simplest explanation to account for this overall pattern of long exon position is a single switch relative to the DD within the deuterostomes after the divergence of echinoderms and before the divergence of tunicates from the vertebrate lineage (Fig 4). The alternate explanation of a separate evolutionary origin for vertebrate and protostome long exons based on position requires loss of one of the longest ORFs in the genome followed by *de novo* insertion of a similarly rare long ORF in a neighboring position, all while maintaining continuity of long exon-containing ankyrins within the phylogeny. In contrast, a switch in the position of an existing long exon would require only a single step of exon shuffling.

Another line of argument against a common origin for vertebrate and *Drosophila* giant ankyrins is that they lack homology. We therefore examined the homology of long exon-encoded polypeptide between key bilaterian species to see if homology is shared only between long exons with the same insertion site, which would strengthen the argument for a separate origin. However, we surprisingly did not find significant homology between mouse AnkG and long exon polypeptides from the deuterostome invertebrates *Ciona intestinalis* and *S*. *purpuratus*, species which have a single ankyrin gene and bracket the switch in long exon position (S2 Fig). Since it is almost certain that the single ankyrin gene in *Ciona* and AnkB and AnkG in vertebrates, all of which share the same long exon insertion site, have a common ancestor, we conclude that the long exon sequence has evolved too rapidly for homology to be a useful indicator for common evolutionary origin. In support of this, there was no detectable homology shared between long exon polypeptides from *Drosophila*, *C*. *elegans*, or *S*. *purpuratus*, which share a common insertion site downstream of the DD, but represent deeply diverging bilaterian phyla (S2 Fig).

**Fig 5:**
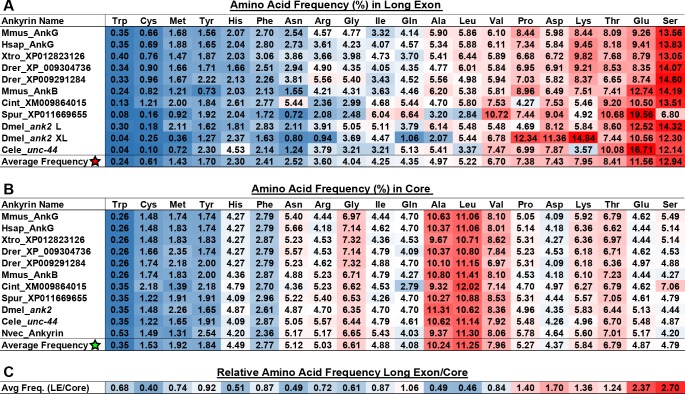
Amino acid composition of ankyrin long exon polypeptides. (A) Amino acid compositions for 11 long exon-encoded polypeptides from 10 bilaterian ankyrin genes. Gene names are given at the left margin, and frequencies are given as percent composition for each amino acid. Frequencies are color coded with a blue (low) to red (high) and amino acids are arranged left to right in ascending order of average frequency. (B) An identical amino acid composition analysis as shown in (A) is given for the core ankyrin motifs (24 ankyrin repeats + the ZU5-ZU5-UPA cassette). Note the very different amino acid enrichment profiles (red highlights). For instance, Ala and Leu replace Glu and Ser as the top two most frequent residues. *Nematostella* ankyrin was included in the core analysis to demonstrate that the composition bias of the core has not changed since the divergence of cnidarians and bilaterians, likely soon after emergence of the first ankyrins. (C) Fold enrichment for each amino acid in long exon vs. core polypeptides, color coded blue (depleted) to red (enriched). The top 6 long exon amino acids are all enriched relative to the core with a > 2-fold enrichment for Glu and Ser. Fold enrichment was calculated for sequence sets shown in A and B by dividing the average amino acid frequency encoded in long exons (A, asterisk) by the average amino acid frequency found in the core (B, asterisk).

Although sequence homology seems to be rapidly lost, we found that long exon polypeptides share in common a strong amino acid composition bias with just two amino acids, Ser and Glu, accounting for approximately 25% of the polypeptide and the top six amino acids (Ser, Glu, Thr, Asp, Lys and Pro) accounting for >55%, even though they represent only 32.8% of all possible codons (Fig 5A). The coding bias was present in long exons with or without repeat arrays. The Ankyrin-ZU5-ZU5-UPA core has a very different composition bias centered on amino acids common in the ankyrin repeats (Ala, Val, Leu), while the most abundant 6 long exon amino acids account for only 31.8%,similar to their codon frequency (Fig 5B). The exact functional significance of such a coding bias is unclear but it does suggest some level of commonality for giant isoform function and it is the most conserved feature of ankyrin long exon sequence. Based on the presence of unusually long exons that encode polypeptides with very distinctive amino acid composition in all bilaterian groups, we conclude that giant ankyrins most likely evolved in a common ancestor to all bilaterians.

### The *Drosophila* Ank2 L exon includes sequences that enrich it at the proximal axon

We hypothesized that if giant ankyrins have a common origin early in bilaterian evolution, then perhaps their function in the organization of the proximal axon might also be widespread across bilaterians. To test this hypothesis, we chose *Drosophila* dendritic arborization (da) sensory neurons as a model system to test ankyrin function. These cells are multipolar sensory neurons that extend their dendrites over the surface of the larva [52]. They are responsible for sensing contractions of the body wall to mediate coordinated movement [53], for nociception [54] and light avoidance [55]. These multipolar neurons lie directly under the cuticle and epidermal cells while the axons dive below the surface and extend to the central nervous system where they make synapses in the ventral ganglion. The dendrites, cell body and proximal axon are thus accessible for live imaging in intact larvae [39, 56]. Moreover, the same cell can be reproducibly labeled and identified in every animal [52] and the organization of the microtubule cytoskeleton is similar in these peripheral neurons and *Drosophila* central neurons [39]. For most experiments we used the ddaE neuron, one of the two dorsal class I neurons, which are responsible for sensing contractions of the body wall to mediate coordinated movement [53].

**Fig 6:**
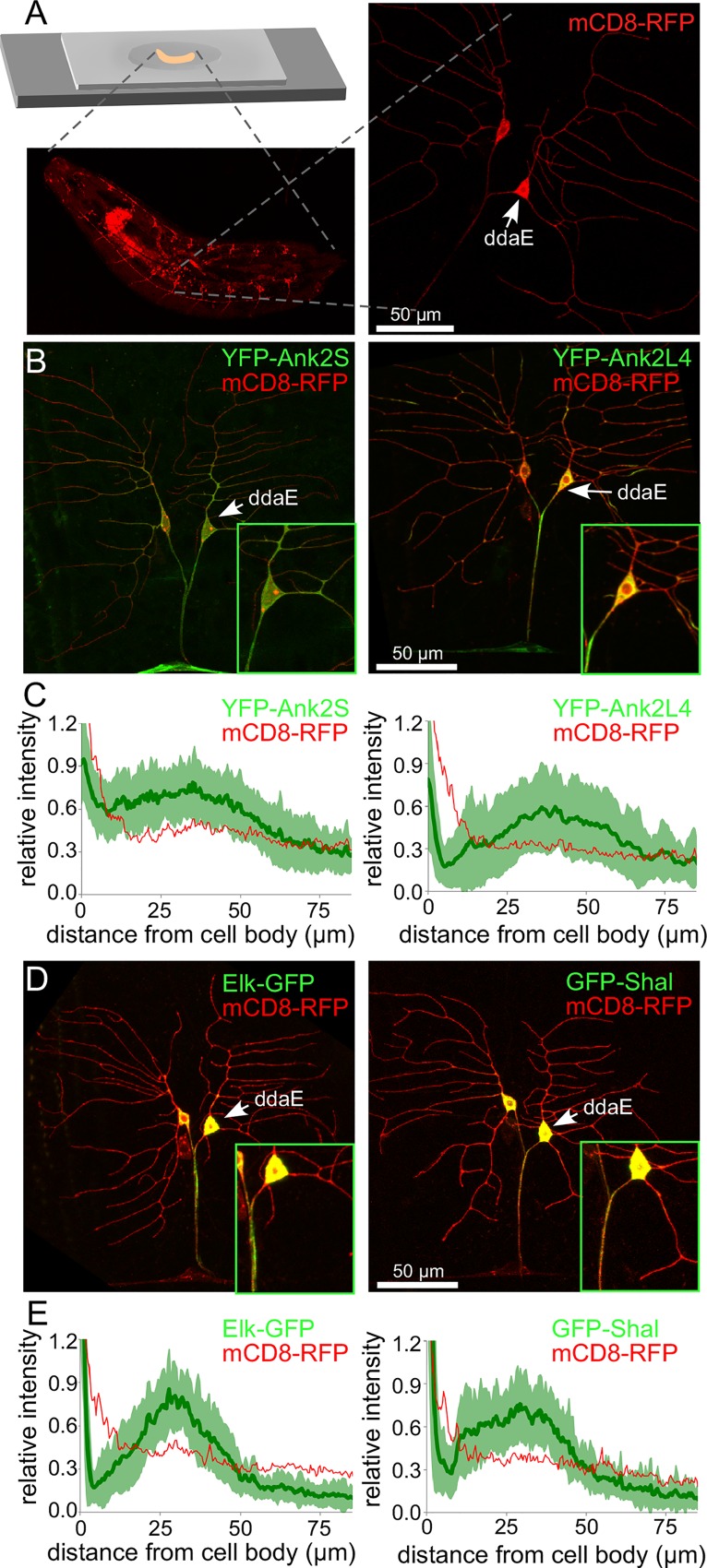
Enrichment of a *Drosophila Ank2L* polypeptide and two tagged ion channels in the proximal axon. A. The strategy to image tagged proteins in whole *Drosophila* larvae is shown. Larvae are placed on a drop of agarose dried onto a slide and held in place by a coverslip taped onto the slide to prevent movement (top left). Neurons on the dorsal side of the animal can be visualized through the cuticle (lower left); the dendrites, cell body and proximal axon of the ddaE neuron are all easily visible (right). B and D. YFP-Ank2S, YFP-Ank2L4, Elk-GFP and GFP-Shal were expressed in class I neurons using 221-Gal4. mCD8-RFP was also expressed to outline the cells. The two dorsal class I neurons are shown, with ddaE at the right and ddaD on the left. Insets show the cell body, proximal axons and proximal dendrites of ddaE. C and E. To quantitatively assess marker distribution, the fluorescence intensities were measured from the cell body along the axon. Averages from 30 cells are shown for each marker. For the Ank2 constructs, and tagged Elk and Shal, the standard deviation is shown as light green around the dark green average line. For mCD8-RFP only the average is shown for comparison.

We focused on examining the role of *Ank2*, because it is the only *Drosophila* ankyrin capable of encoding giant isoforms, it is enriched in neurons [57], and giant Ank2 isoforms have other demonstrated roles in axon function [15, 16]. When a protein containing YFP fused to aa1-1159 (Ank2S) [15], which includes only the ankyrin repeats and the first ZU5 domain, was expressed in class I neurons, fluorescence extended out from the cell body quite evenly into the dendrites and axon, gradually tapering in both compartments with increasing distance (Fig 6A and S3 Fig). In contrast, YFP-Ank2L4, YFP fused to an Ank2 fragment that includes the first half of the L exon (aa1530-3005 of the Ank2-LP protein on Flybase) including a microtubule binding domain [15], was concentrated in the part of the axon immediately adjacent to the cell body and very little was present in dendrites (Fig 6B and 6C, S3 Fig). The distinct distribution pattern of Ank2L4 in the proximal axon is clear when compared to the general membrane marker mCD8-RFP, which was used to outline the cells (Fig 6C). This ability of part of the polypeptide encoded by the L exon to concentrate in the proximal axon suggested that *Drosophila* giant ankyrins may well localize to the proximal axon in addition to distal axonal regions as has been reported for motor neurons [15, 21].

### Elk and Shal K^+^ channels are enriched in the proximal axon

We expressed two voltage-gated K^+^ channels fused to GFP, Elk-GFP [40] and GFP-Shal [58], in ddaE neurons in order to see whether ion channels can also accumulate within the proximal axon. These two K^+^ channels have previously been shown to accumulate within the proximal axon of mushroom body γ-neurons [40]. In mammalian neurons, Shal channels (Kv4) regulate spike shape and patterning [59] while Elk channels regulate firing threshold [60]. Both Elk-GFP and GFP-Shal concentrated within the beginning of the axon of ddaE neurons (Fig 6D and 6E, S3 Fig), overlapping the proximal side of the AnkL4-YFP peak. The proximal axon of ddaE neurons thus appears to have an ability to accumulate membrane proteins such as ion channels.

### A plasma membrane protein has reduced mobility in the proximal axon

**Fig 7:**
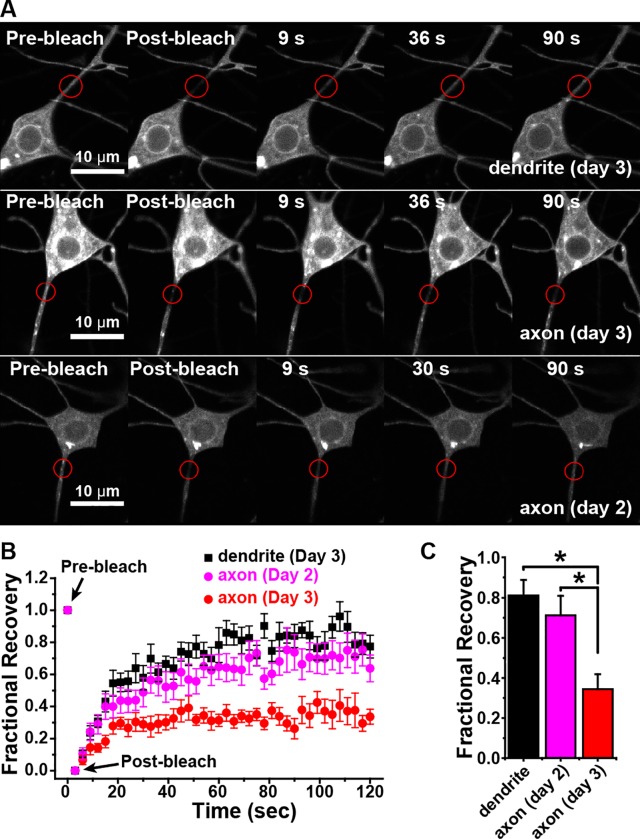
Diffusion in the plasma membrane is restricted at the base of the axon compared to the base of the dendrite. A. Example images of dendrite and axon FRAP experiments at two different larval ages are shown. mCD8-RFP was expressed in class I dendritic arborization neurons. Bleaching was performed in the ddaE neuron either at the base of the comb-like dendrite or the base of the axon. The red circle indicates the bleach area. B. The average recovery of fluorescence into the bleach areas as shown in A is plotted on the graph; averages were calculated from 14 cells for the dendrite, 17 for axon 2 day and 13 for axon 3 day. Each cell was in a different animal. The error bars show the standard error of the mean. C. Bars show the recovery plateau (mean ± SEM) quantitated by averaging recovery values between 110 and 120 ms. The asterisk indicates significant difference (P < 0.01, t-test).

**Fig 8:**
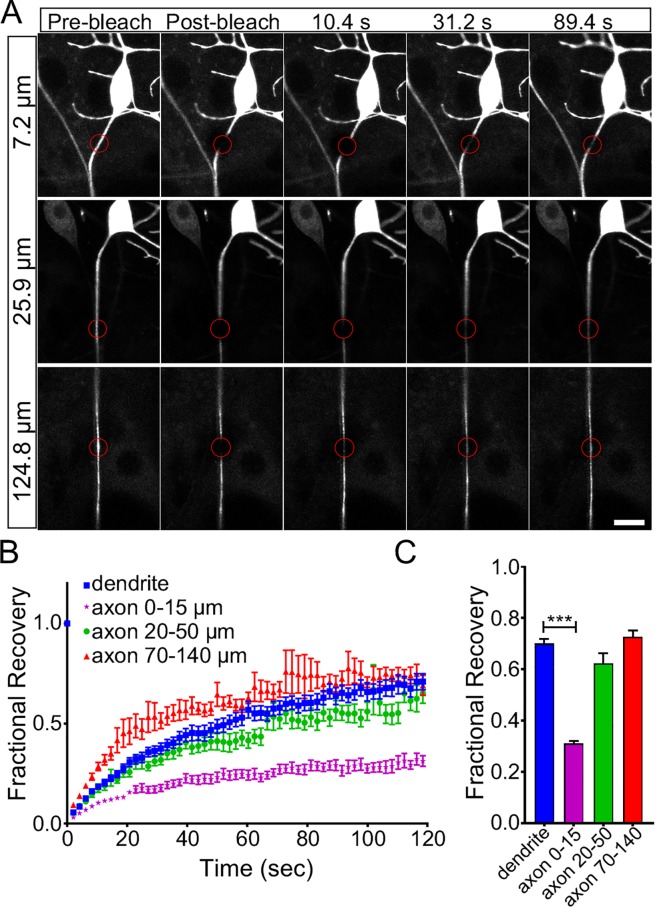
The axonal diffusion barrier is in the first 20 microns of the ddaE neuron. A. mCD8-GFP was expressed in the ddaE neuron, and examples of FRAP experiments are shown. For the proximal axon region the ddaD cell was not killed, but for the more distal regions the neighboring ddaD cell was killed so that signal from ddaE could be collected without interference from a bundled axon. The bleach area is indicated by the red circle, and the distances indicated are the distance from the start of the axon to the side of the bleach region closest to the cell body. B. The average recovery of fluorescence into the bleached area was plotted. The n for each group was: 15 for dendrite, 25 for axon 0–15 μm, 16 for axon 20–50 μm, 13 for axon 70–140 μm. The error bars show the standard error of the mean. C. The recovery plateau; the average values between 110 and 120 seconds, for each group are shown. *** p value <0.001 calculated with an unpaired t-test. Error bars show the standard deviation.

We hypothesized that if giant ankyrins play a role in plasma membrane protein tethering in the proximal axon of ddaE, then there should be an ankyrin-dependent plasma membrane diffusion barrier similar to that found in vertebrates. We expressed mCD8-GFP or mCD8-RFP in ddaE neurons to examine diffusion in the plasma membrane. mCD8-GFP is diffusible membrane marker used extensively in *Drosophila*. In the cell body mCD8-RFP (Fig 7) and mCD8-GFP (Fig 8) localize to both the ER and plasma membrane, but in the axon they are primarily at the plasma membrane and occasional internal vesicles (Figs 7A and 8A). In a previous study mCD8-GFP was used to identify a plasma membrane diffusion barrier half way out developing axons of cultured neurons, and behaved similarly to other membrane markers including ROBO-GFP and a GPI-anchored GFP [41]. Here, we looked for diffusion barriers in axons in living animals for the first time. We first used fluorescent recovery after photobleaching (FRAP) to compare the mobility of mCD8-RFP in the proximal axon and dendrites, as no diffusion barrier has ever been described at the base of the dendrite. FRAP experiments were performed in whole, living 3-day old larvae. To normalize the results, the fluorescence intensity before bleaching was set to 100% and after bleaching it was set to 0. Bleaching occurring during imaging itself was accounted for by taking the ratio of the fluorescence intensity in the bleached area and the fluorescence intensity in an unbleached area of the soma. Occasional blips in fluorescence occurred when vesicles trafficked through the axon. At the dendrite base, mCD8-RFP fluorescence routinely recovered to greater than 70% initial intensity by 60s, and by 90s it was often difficult to tell where the bleach region had been (Fig 7A). In contrast, fluorescence in the proximal axon recovered to only about one third of the original intensity, and the bleach region could still be clearly seen at the end of the time course (Fig 7A). For a direct comparison of recovery in the axon and dendrite, we performed simultaneous bleaching in both regions and made a movie of the FRAP experiment (Movie 1). Fluorescence very obviously recovers into the dendrite bleach area, while recovery into the axonal bleach area is very limited. Comparisons of the recovery plateau revealed that fluorescence recovery was significantly suppressed in axons relative to dendrites (Fig 7B). This limited diffusion was not a general property of the axon as FRAP assays using an ER membrane marker Rtnl1-GFP (protein trap G00071 [61]) showed rapid recovery in the proximal axon at the same time point (S4 Fig). We conclude that diffusional mobility of plasma membrane proteins is reduced in the proximal axon relative to the proximal dendrite.

In cultured mammalian neurons, the AIS diffusion barrier is established at 7–10 days, well after axons and dendrites become morphologically distinct and take on their distinct microtubule organization [3, 10], which occurs between 4–7 days [62]. In *Drosophila* dendritic arborization neurons, axons emerge first during embryogenesis, followed by dendrites, and dendrite microtubule polarity becomes mature by the second day of larval life [63]. To test whether the diffusion barrier was in place by the time these other aspects of polarity emerged, we performed FRAP analysis of mCD8-RFP in the proximal axon of two day old larvae. At this time point, recovery was similar to that in dendrites (Fig 7) indicating that, as in cultured mammalian neurons, restricted diffusion in the proximal axon is a relatively late polarized feature to develop.

To determine whether limited diffusion represents a proximal axon diffusion barrier or is simply a generalized property of ddaE axons, we next performed FRAP experiments bleaching mCD8-GFP in the proximal axon (0–15 μm from the cell body) as in Fig 7, 20–50 μm from the cell body (roughly within the Ank2L4 peak), or 70–140 μm from the cell body (in the distal axon shaft). Measurements at the two distal sites were conducted 24h after ablating the neighboring ddaD neuron as its axon bundles with the ddaE axon ~25 μm from the cell body. We found that the diffusion barrier was limited to the proximal site 0–15 μm from the cell body; recovery of mCD8-GFP fluorescence was not significantly reduced relative to dendrites at either the middle or distal bleaching sites (Fig 8A–8C). ddaD was not laser-ablated for bleaching at the proximal axon site for data shown in Figs 7 and 8, but we confirmed that the diffusion barrier seen in the proximal axon remained when ddaD was ablated (S5 Fig).

### Ank2L is required to restrict plasma membrane diffusion in the proximal axon

**Fig 9:**
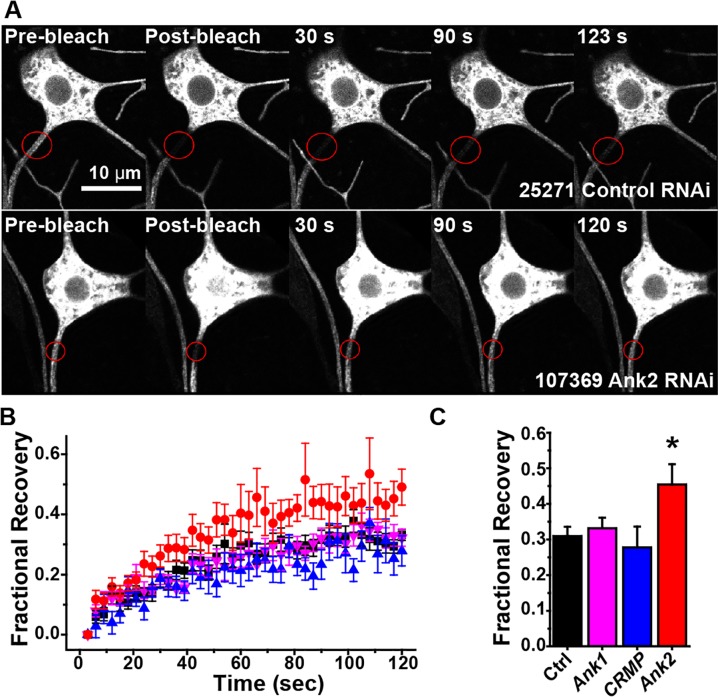
RNAi targeting Ank2L reduces the axonal diffusion barrier. A. mCD8-GFP was expressed in class I neurons, and proximal axons of ddaE neurons were photobleached. Example images of a control neuron are shown in the top row, and Ank2 RNAi neurons below. B. Quantitation of FRAP experiments in different genetic backgrounds is compiled in the graph. The average recovery is shown, with standard error shown as error bars. Number of cells tested for each genotype were: control-24, Ank2-19, Ank-12, CRMP-9. C. The recovery plateau (mean ± SEM) was quantitated by averaging recovery values between 110 and 120 ms. The asterisk indicates significant difference (p < 0.02, t-test).

Having shown that the axon diffusion barrier is restricted to a region proximal to the peak of Ank2L4 localization (Fig 6C), we wished to determine whether the barrier indeed depended on Ank2 long exon-containing isoforms (Referred to here as Ank2L). To reduce neuronal levels of Ank2 in animals in which development was otherwise normal, we expressed large RNA hairpins specifically in class I neurons with the Gal4-UAS system. This has been demonstrated to be an effective way to perform cell-type specific RNAi in *Drosophila* [64]. Hairpins are several hundred nucleotides long and are processed into dozens of different short hairpins that trigger destruction of their target. As a control we expressed a hairpin targeting γ-tubulin37C, a maternal gene that is not expressed in somatic cells [65]. In addition to the hairpin RNAs, mCD8-GFP and dicer2 were expressed in class I neurons. When an Ank2L-directed RNA hairpin was expressed in ddaE, mCD8-GFP fluorescence recovered to a higher plateau into the proximal axon after photobleaching than with control hairpins or hairpins targeting Ank or CRMP (Fig 9). The Ank2 hairpin used contains a sequence at the 3’ end of the L exon and thus specifically targets transcripts containing the L exon. Because the RNA hairpins are expressed in only a few cells in the animal compared to Ank2 itself, it is difficult to determine level of knockdown using qPCR, however the increased recovery specifically when Ank2 was targeted suggested that its protein levels were reduced and that its function is important for limiting diffusion in the proximal axon (Fig 9B and 9C). We could not make any conclusions about the role of CRMP or Ank in the absence of verification of knockdown because they did not show a phenotype.

**Fig 10:**
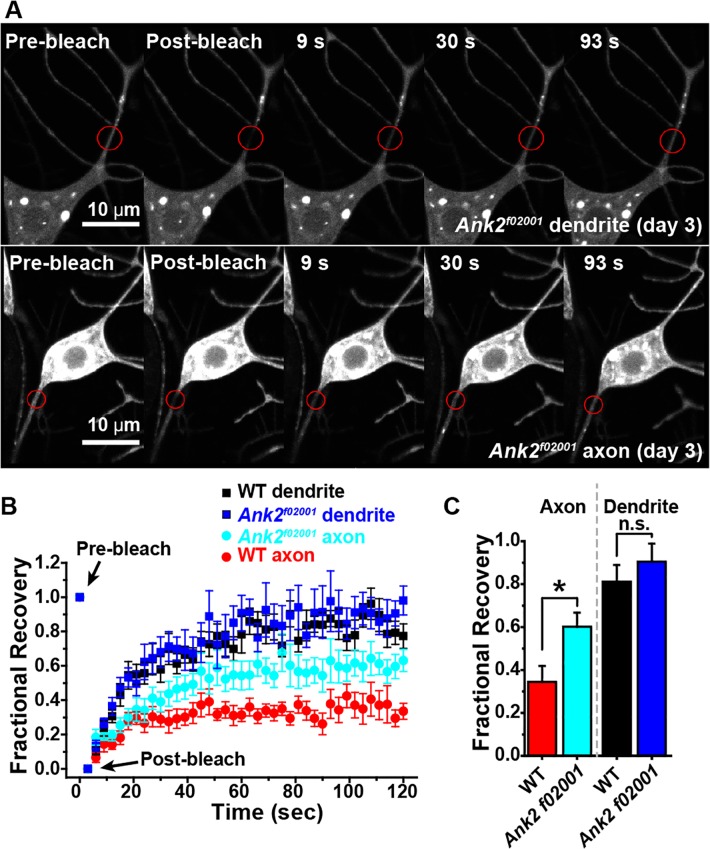
Disruption of the Ank2L exon compromises the axonal diffusion barrier. A. Example images of dendrite and axon bleaching experiments in *Ank2*^*f02001*^ homozygous animals are shown. The fluorescent marker is mCD8-RFP as in Fig 7. B. Quantitation of FRAP experiments is shown. The control data is the 3-day dataset shown in Fig 7. This is the matched control for the *Ank2*^*f02001*^ mutants as the age of the animal, Gal4 driver and fluorescent marker are the same. N’s for the control are listed in the legend for Fig 7. For the mutant, FRAP was measured in dendrites of 9 cells and axons of 19 cells. C. The recovery plateau was quantitated by averaging values between 110 and 120 ms. Bars show mean ± SEM. and the asterisk indicates significant difference (p < 0.02, t-test).

To more rigorously test a specific requirement for giant Ank2, we used a previously characterized P element insertion that disrupts the L exon [15, 16]. We generated *Drosophila* larvae in which both copies of the L exon contained the f02001 insertion and mCD8-RFP was expressed in class I neurons. Diffusion in the ddaE proximal dendrite was similar to control neurons (Fig 10), but mCD8-RFP recovered to a significantly higher level after photobleaching in proximal axons of *Ank2*^*f02001*^ mutant neurons than in control neurons (Fig 10). Axonal membrane diffusion in the *Ank2*^*f02001*^ did not fully recover to dendritic levels, which could be due to incomplete removal of relevant long ankyrin isoforms by the *Ank2*^*f02001*^ mutation [15], leftover maternal contribution of Ank2, or ankyrin-independent mechanisms. The significant increase in diffusion in the *Ank2*^*f02001*^ mutant neurons strongly suggests that giant isoform(s) of Ank2 containing the L exon are required to establish a diffusion barrier in the proximal axon.

### Shal K^+^ channel accumulation in the proximal axon depends on Ank2L

**Fig 11:**
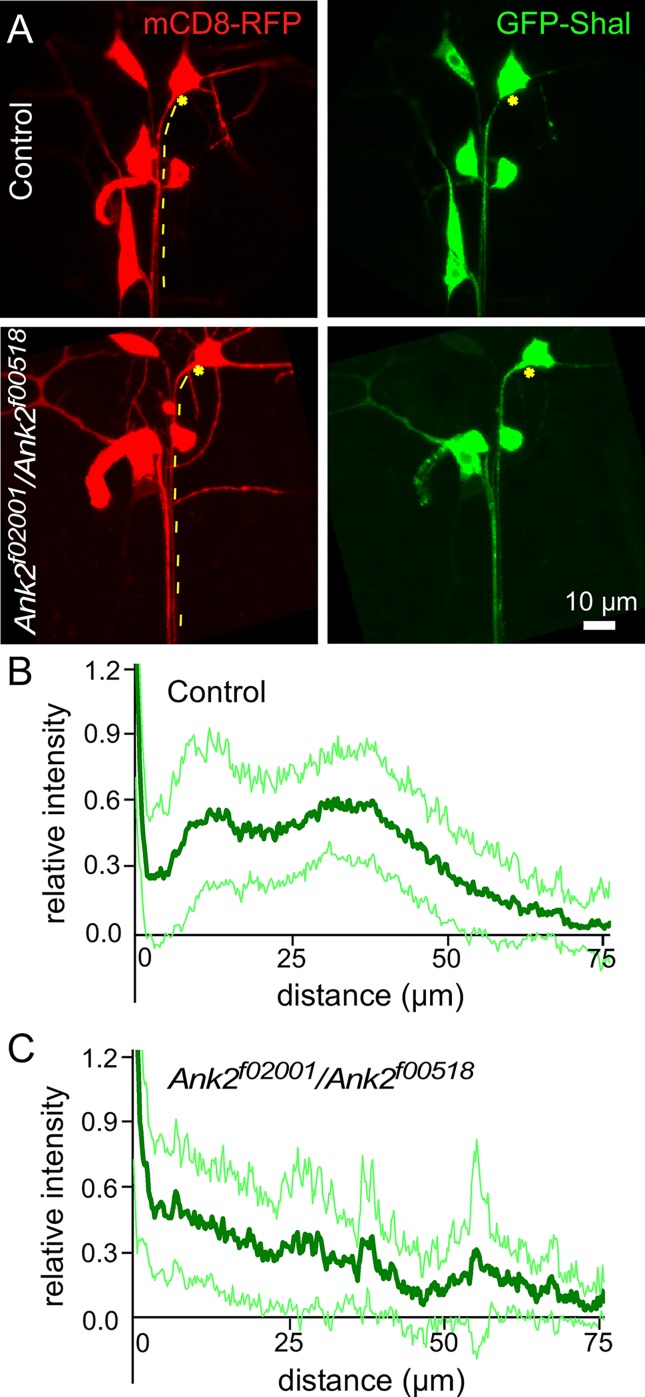
GFP-Shal patterning in the proximal axon depends on Ank2. A. Examples of GFP-Shal localization in control or *Ank2*^*f02001*^*/Ank*^*f00518*^ are shown. mCD8-RFP was expressed to outline the cell. Stars mark the beginning of the axon and dashed lines trace the ddaE axon. B and C. Quantitation of GFP-Shal in control and of *Ank2*^*f02001*^*/Ank*^*f00518*^ neurons was performed by measuring the fluorescence intensity along the axon starting from the cell body until it was no longer possible to separate the signal in the ddaE axon from that of neighboring axons. Because of this overlap with other axons, n’s were not the same at all distances: n’s for the control group are 52, 52, 44, 32 at 0, 25, 50 and 75 microns away from cell body, and n’s The for the *Ank2*^*f02001*^*/Ank*^*f00518*^ group are 36, 29, 18, 8 at 0, 25, 50, 75 microns away from cell body. Scale bar = 10 microns. Average normalized fluorescence intensities are shown as the dark green line and the standard deviation is represented by the pale green surrounding lines.

We next examined whether GFP-Shal accumulation within the proximal axon depends on Ank2L. We used a *Ank2*^*f02001*^*/Ank2*^*f00518*^ trans-heterozygous background to reduce Ank2L; *Ank2*^*f00518*^ contains a p-element insertion in the common ankyrin repeat region and blocks expression of all Ank2 isoforms [15]. The peak accumulation of GFP-Shal occuring between ~10–50 μM from the cell body (Fig 11A and 11B) was greatly reduced in *Ank2*^*f02001*^*/Ank2*^*f00518*^ larvae (Fig 11A and 11C). In the mutant background, axonal GFP-Shal concentration is highest immediately adjacent to the cell body and simply decays in concentration with distance. The accumulation of GFP-shal 10–50 μM from the cell body therefore appears to depend on Ank2 expression.

## Discussion

To assess whether giant ankyrins exist and function in the proximal axon outside the vertebrate lineage we performed extensive analysis of metazoan ankyrin genes to determine where complete ankyrins were present, and then determined which of these contained long exons that could potentially encode giant ankyrins. In parallel to this phylogenetic approach, we performed functional analysis to determine whether *Drosophila* neurons have a diffusion barrier in the proximal axon, and whether this depends on the *Drosophila* giant ankyrin. In contrast to the prevailing view in the field, we found evidence to support a single origin for giant ankyrins early in bilaterian evolution. This finding was previously obscured by rapid sequence divergence in the long exons included in giant isoforms. With the increased availability of transcriptome and genome data from phylogenetically diverse eumetazoans, we could circumvent this sequence homology limitation to trace the evolutionary origins of the long exons by their presence or absence and position. This analysis allowed us to determine that long exon-encoded giant ankyrins are the rule, not the exception, in bilaterians. We therefore propose a revised view of ankyrin evolution in which giant ankyrins have a single common evolutionary origin in ancestral bilaterians prior to the radiation of extant bilaterian phyla. While we do not know exactly when these giant ankyrins gained the ability to organize the AIS, the fact that they are required for diffusion barrier formation in the proximal axon in both mammals (deuterostome) and *Drosophila* (protosome) suggests that this function may be an ancestral feature of giant ankyrins.

Drosophila Ank2 is unusual (though not unique) in the it has two long exons (L and XL) and can thus encode diverse giant isoforms. In this study we focused on disruption of isoforms that include the L exon to show that these giant isoforms are required to set up an axonal diffusion barrier and to localize a K^+^ channel marker (GFP-Shal) to the proximal axon. Because loss of L exon-containing isoforms is known to also disrupt localization and expression of XL exon-containing isoforms [16, 21], it is not currently possible for us to determine whether L, XL or both L and XL isoforms are required for the diffusion barrier and GFP-Shal localization in the proximal axon.

The diffusion barrier we found occurs proximal to the peak of the Ank2L4 marker accumulation we observed in the axon, but the barrier nevertheless depends on Ank2. This appears at odds with the model for cultured mammalian neurons that it is the density of membrane proteins attached to AnkG that forms the diffusion barrier [3]. However, it is important to note that the Ank2L4-GFP marker probably does not reflect the complete distribution of ankyrins within the proximal axon, and it is therefore possible that native ankyrin isoforms are present in the diffusion barrier region between the observed Ank2L4-GFP peak and the cell body. AIS patterning in cultured mammalian neurons is entirely dependent on giant AnkG, but not all membrane proteins concentrated in the AIS bind directly to AnkG. If the AIS is similarly complex in ddaE neurons, it alternatively could be that the diffusion barrier is formed by a protein-dense membrane region separate from (but held in place by) a neighboring giant Ank2-rich region. In cultured mammalian neurons, diffusion is limited in the AnkG-rich region [3]. However, membrane diffusion in mammalian axons has not been examined proximal to the cell body equivalent to where we find the barrier in ddaE neurons [2, 3], so the extent of overlap between diffusion barrier and AnkG is not fully known and a situation similar to that in ddaE neurons cannot be ruled out.

The AIS as defined in mammalian neurons includes not only a special submembrane cytoskeleton organized by giant ankyrins and a plasma membrane diffusion barrier, but also a concentration of ion channels that control action potential initiation. While we did not look directly at native ion channel localization in this study, we showed that two potassium channels, Elk and Shal, can accumulate in the proximal axon and that the accumulation of Shal is dependent on Ank2. We think it is likely that native ion channels will be found to accumulate in the proximal axon of ddaE neurons, as it is the concentration of membrane proteins including ion channels and cell adhesion molecules orchestrated by giant ankyrins that is believed to be the primary contributor to limited plasma membrane diffusion [3, 8, 10].

In a study focusing on the subcellular organization of Drosophila mushroom body γ neurons, a type of kenyon cell interneuron in the brain, a variety of different markers, including channels and cytoskeletal proteins were found to have specific localization patterns within the axon. Several markers including ankyrin, Elk and Shal were enriched in the proximal axon, and others were present only on one side of a boundary region [40]. Localization of one of this latter class of proteins, Fas2, was sensitive to actin depolymerization [40], which disrupts the AIS in mammalian cells [2]. The presence of a diffusion barrier and role of giant ankyrins in setting up these localization patterns was not examined. One point to note is that the kenyon cells are unipolar, so the specialized region is not adjacent to the cell body as in typical mammalian neurons, but is instead found further out the neurite, past the point where dendrites branch out. The multipolar geometry of dendritic arborization neurons used here is more similar to that of the mammalian neurons in which the AIS has been studied, and in this setting the diffusion barrier is next to the cell body as it is in mammals.

The question of which native ion channels are clustered in the proximal axon of *Drosophila* neurons is an important one, and the answer will shed light on how and when the proximal axon evolved as the site of action potential initiation. Insect Na^+^ channels do not have the AnkG binding domain found on vertebrate neuronal Na^+^ channels. This binding site interestingly evolved early in the deuterostome lineage before the appearance of AnkG [12]. Our results now show that giant ankyrins were also present at this time, raising the possibility that giant ankyrins were mediating axonal Na^+^ channel clustering long before the evolution of vertebrates. While it is clear that the particular ankyrin binding domain found on vertebrate Na^+^ and KCNQ K^+^ channels is not conserved across the bilateria, this should not be taken as evidence to rule out an ancient, conserved role for ankyrins in clustering ion channels at the AIS or other axonal locations. A crystal structure of the ankyrin repeat array of a vertebrate ankyrin suggests that binding ankyrin domains could be highly diverse and probably cannot be predicted from primary sequence [66]. Furthermore, some mammalian AIS proteins such as Kv1 channels do not directly bind to AnkG [67], but nevertheless show AnkG-dependent AIS localization [68], presumably due to the central role AnkG plays in initiating AIS patterning. Therefore, the absence of this one ankyrin binding motif does not provide definitive evidence against ankyrin-dependent localization to the proximal axon. One can envision a scenario in which ankyrin-based tethering of channels in the axon and/or diffusional exclusion of somatodendritic channels evolved in ancestral bilaterians to facilitate axonal action potential initiation, but needed further refinement in vertebrates to accommodate the spatial constraints imposed by myelin. These refinements may have altered the composition of AIS-clustered channels over time. It will likely be necessary to experimentally determine a comprehensive map of channel localization in the proximal axon of neurons from key bilaterian model species such as *Drosophila* before reaching conclusions on how AIS function evolved within the bilateria. However, if organization of the plasma membrane proteins at the AIS is exclusively dependent on giant ankyrins, then it is unlikely that a specialized ankyrin-based AIS predates the bilaterians as we find no evidence for giant ankyrins in cnidarians or placozoans.

The results we present here suggest that giant ankyrins have a common origin in a bilaterian ancestor, and that they may play an evolutionarily conserved role in patterning of the axon initial segment in protostomes and deuterostomes. These data require revision of the current model of axon evolution which places the giant ankyrin-based AIS as a relatively recent vertebrate-specific innovation. Further characterization of molecular architecture of axons in invertebrate model organisms will be needed to determine which features of the vertebrate AIS are indeed new innovations and which features are ancient and widely shared among bilaterians.

## Materials and Methods

### Ankyrin gene identification and characterization

Ankyrin genes in genomes, genome annotations and transcriptomes could be reliably identified with TBLASTN [69] using mouse AnkG and *Drosophila* Ank2; these baits yielded an identical set of ankyrin hits in all databases tested. We first used only the ZU5-ZU5-UPA cassette of mouse ANKG and *Drosophila Ank2* as queries for the TBLASTN searches because this cassette is specific to true ankyrin genes, whereas ankyrin repeats can be found in a wide range of proteins including several classes of TRP family ion channels [70, 71]. Hits were verified as ankyrins if 1) they had the canonical ankyrin repeat cassette upstream of the ZU5-ZU5-UPA cassette, and 2) they had reciprocal best matches to the three mouse ankyrin orthologs when used as TBLASTN queries against the mouse RefSeq database. Gene predictions were manually adjusted if they had gaps in highly conserved regions and we were able to find the corresponding missing exon in the genome draft. Giant isoform predictions and transcripts were mapped to the corresponding genome draft to verify the presence of a long exon(s) and determine its position within the gene and boundaries. When necessary (*Ciona intestinalis* for example), the presence or absence of potential long exons was determined by manually searching the ankyrin gene locus for ORFs between the UPA domain and the adjacent gene.

### *Ciona* Ankyrin long exon PCR

5’ and 3’ junctions of the *Ciona intestinalis* Ankyrin large exon were verified by PCR of cDNA from whole larvae. A PCR product spanning the 5’ long exon junction was amplified in two rounds using the following primers for the first round: 5’-TTAACATTCTACAAACTTCCACTGG-3’ (sense) and 5’-GAGGCCTCTTCTTTAATTATCACTTC-3’ (antisense). A faint ~2.7 Kb band from this reaction was gel purified and amplified with the same sense oligo and a nested antisense oligo (5’- ATGGTTTGGAGAATCAGGTGAG-3’) to obtain an ~ 1.6 Kb band sufficient for cloning. An ~ 1Kb band spanning the 3’ junction was obtained using the same strategy; First round primers were 5’-CTGTTTCACCTGGTTTATCCCGTAGC-3’ (sense) and 5’-GCACAAATCTCCTCGACCAATACTAT-3’ (antisense), and in the second round the sense primer was replaced with 5’- ACTTCACGGTCATGCAACTGCTCCTT-3’. Both products were cloned and sequence verified to determine the Ciona ankyrin giant isoform sequence given in S2 Table.

### ORF analysis

ORF sizes were calculated to determine rank of ankyrin long exon size for the following genome drafts: *Mus musculus*, GRCm38/mm10, (ftp://hgdownload.cse.ucsc.edu/goldenPath/currentGenomes/Mus_musculus/bigZips/); *Ciona intestinalis*, Mar. 2005 freeze, (ftp://hgdownload.cse.ucsc.edu/goldenPath/currentGenomes/Ciona_intestinalis/bigZips/); *Drosophila melanogaster*, Aug. 2014 (BDGP Release 6 + ISO1 MT/dm6) (ftp://hgdownload.cse.ucsc.edu/goldenPath/currentGenomes/Drosophila_melanogaster/bigZips/); and *Caenorhabditis elegans*, UCSC version ce6 (ftp://hgdownload.cse.ucsc.edu/goldenPath/currentGenomes/Caenorhabditis_elegans/bigZips/). ORFs were identified using a custom Python script, which assumed a minimum ORF size of 100 amino acids free of stop codons, and also filtered out regions that would be interrupted by long stretches of simple repeats. The script is publicly available at https://github.com/bishoyh/ORFeome-calculator. Statistics were calculated using Mathematica version 10.3 (Wolfram, Champain, IL).

#### Phylogeny

Ankyrin sequences were trimmed to include only the 24 ankyrin repeats and the ZU5-ZU5-UPA cassette and aligned for phylogenetic analysis using MUSCLE as implemented in MEGA6 [72] and optimized manually as necessary. Linker regions lacking length conservation were trimmed. The phylogeny was determined in MEGA6 using Maximum Likelihood methods with an LG substitution model and 5 discrete Gamma categories and tested with 100 Bootstrap replications.

#### *Drosophila* Stocks and Genetics

Many transgenic *Drosophila* lines were obtained from the Bloomington *Drosophila* Stock Center (http://flystocks.bio.indiana.edu/) and the Vienna *Drosophila* RNAi Center (http://stockcenter.vdrc.at/), which are invaluable resources. Dr. Tadashi Uemura provided IGI1-Gal4. Dr. Susan Tsunoda provided UAS-GFP-Shal flies and Dr. Julie Simpson provided the UAS-Elk-GFP flies. UAS-Rtnl1-GFP was from the FlyTrap project [61].

To visualize the plasma membrane in the ddaE neuron UAS-mCD8-GFP was expressed with 221-Gal4, or UAS-mCD8-RFP was expressed with IGI1-Gal4. For visualizing tagged Ank2, transgenic lines containing different portions of the coding region fused to the GFP coding sequence were kindly provided by Dr. Jan Pielage (described in [15]). These were crossed to a line containing 221-Gal4 and UAS-mCD8-RFP to generated larvae to image.

For RNAi experiments, the tester line UAS-dicer2; 221-Gal4, UAS-mCD8-GFP was crossed to RNA hairpin lines and larval progeny were analyzed. The RNAi lines used were: Ank2 RNAi (VDRC #107369), Ank RNAi (VDRC #25946), and the control used was γ-tubulin37C RNAi (VDRC #25271). Imaging of RNAi larvae was performed after collecting embryos for 24h and then aging the animals for 3 days at 25°C. For experiments with *Ank2* mutants, lines were constructed with IGI1-Gal4, UAS-mCD8-RFP on the second chromosome and the *Pbac[WH]Ank2*^*f02001*^ insertion balanced with TM6 on the third chromosome. This line was crossed to *Pbac[WH]Ank2*^*f02001*^*/TM6* to generate homozygous mutant larvae (without the TM6 chromosome, which can be detected in larvae with the Tb, tubby, marker) with one copy of the Gal4 driver and mCD8-RFP. A similar strategy was used to put GFP-Shal in an Ank2 mutant background, except that in this case one of the parental lines contained UAS-GFP-Shal on the second chromosome and *Pbac[WH]Ank2*^*f00518*^*/TM6* on the third chromosome. Matching controls were generated by crossing IGI1-Gal4, UAS-mCD8-RFP animals to wild-type. Embryos were collected for 24h, and then this cohort of larvae was aged for 3 days at 25°C before imaging, except for the one specified instance where 2 day old larvae were tested for the presence of the diffusion barrier. Larvae were therefore 2–3 days old for the 2 day experiments and 3–4 days old for the 3 day experiments.

#### Confocal Microscopy

To perform live imaging, larvae were rinsed in Schneider’s medium or PBS and placed on a dried agarose pad on a glass slide, dorsal side up, and a cover slip was taped on top. Larvae were viewed immediately after mounting on a Zeiss LSM510 or LSM700 microscope or Olympus FV1000 confocal microscope GFP and YFP were excited using a 488 nm laser, and RFP with a 563 or 543 nm laser. Images were acquired using a 63X 1.4 NA oil objective lens and analyzed using ImageJ (http://rsbweb.nih.gov/ij/). Fluorescence intensity of YFP-tagged Ank2 polypeptides and tagged channels was measured in ImageJ starting from the cell body with a line drawn along the axon. Averages and standard deviations for 30 cells from each genotype were measured. The intensities along the axon were normalized first by dividing each value by the average of the lowest 25% of the values, and then this value was normalized to the upper end of the scale by dividing by the average of the highest 5% of the intensity values. This top and bottom normalized intensity is plotted in the graphs.

### Photobleaching experiments

Fluorescence recovery after photobleaching (FRAP) was performed using the scanning confocal microscopes listed above. For each set of experiments shown in a figure the same microscope and conditions were used for controls and experimental samples. Quantification of all movies was done in ImageJ using a plug-in designed to analyze fluorescence intensity in manually picked regions. Bleaching of the whole cell due to imaging over time was accounted for by dividing the bleached region by the unbleached region, and then by dividing the resulting ratio by the initial prebleach ratio to correct for any differences. The results were then normalized to obtain the percentage of fluorescence recovery after bleaching.

For the experiments testing the location of the diffusion barrier along the axon, 5-micron segments were photo-bleached in regions 0–15 μm, 20–50 μm or 70–140 μm from the cell body. Quantification of these FRAP movies was done in ImageJ using a FRAP Calculator macro developed by Dr. Robert Bagnell. One issue for the two regions further from the cell body was that axons from the ddaE neuron that we used for our experiments were bundled together with axons from the ddaD in these areas. To get around any interference from the ddaD axon, this cell was killed 24h before the bleached experiments were performed. Cell killing was accomplished by aiming a MicroPoint pulsed UV laser (Andor) at the ddaD nucleus.

## Supporting Information

S1 Fig Origin of multiple long exons in ankyrin genes. (A) Two long exons in the *Branchiostoma floridiae* (amphioxus, cephalochordate) share a large block of homology (light blue) and thus appear to arise from a duplication of an ancestral long exon. (B) In Zebrafish AnkB, an intron separates two sequence blocks (green and pink), that are contiguous in AnkB sequences from mouse and human, indicating that the two exons arise from a recent intron insertion into a single ancestral long exon. Similar intron insertions appear to have created multiple long exons in ankyrins from the sea urchin *Lytechnicus variegatus* and the horseshoe crab *Limulus polyphemus* (not shown).
(TIF)

S2 Fig Ankyrin long exon sequence conservation. (A) Percent amino acid identity of long exon-encoded polypeptides found in pairwise comparisons. The first long exon polypeptide sequence in each comparison is listed above the colored bar, while the second is listed in the X-axis legend. For mouse AnkG comparisons (red), we also ran controls in which sequence order for the second polypeptide was randomized (black). Data show mean ± S.E.M. of values obtained from 6 different alignment techniques, and asterisks mark values significantly higher than randomized controls (t-test, p < 0.05). The Mouse AnkG long exon shares homology significantly above the randomized control background only with vertebrate AnkG and AnkB orthologs. No significant homology was detected between mouse AnkG and Ciona Ankyrin long exons, which share a common insertion position upstream of the DD. (B) Percent of identities in pairwise alignments generated with the MAFFTWS algorithm occurring as singlets (squares) or in blocks of ≥ 4 consecutive identities (circles). Note that the percent of identities occurring in blocks of ≥ 4 is near zero in randomized controls (black), suggesting that homology blocks of this size are an indicator of true rather than random similarity. In contrast, randomized controls show a high percentage of singlet matches, suggesting that singlet matches in that absence of larger blocks simply represent random background instead of true homology. This homology block analysis agrees with the percent identity analysis in (A) in indicating that the long exon of mouse AnkG only has detectable true homology to long exons from vertebrate AnkGs and AnkBs. All other comparisons show the random control pattern of a high percentage of singlet matches and few if any matches in blocks of ≥ 4. Note both types of comparisons, straight percent identity and homology block analysis, also fail to detect meaningful conservation of long exon sequence between deeply diverging invertebrate species, regardless of exon position.
(TIF)

S3 Fig Grey scale images of the GFP channel for ddaE neurons overexpressing GFP-fusions of Ank2S, Ank2L4, Elk and Shal. The GFP channel is shown in isolation for the dual color images presented in Fig 6B and 6D to allow a clear view of the distribution of the GFP-fusion protein.
(TIF)

S4 Fig FRAP assays targeting the ER membrane marker rtnl-GFP in ddaE neurons show no evidence for an ER diffusion barrier in the proximal axon relative to the proximal dendrite. (A) Time series of images showing recovery from photobleaching of Rtnl1-GFP in the proximal dendrite and axon of a ddaE neuron. The bleached region is circled in red. (B) Quantitation of FRAP experiments in 11 animals each for axon and dendrite bleaching is shown. Error bars indicate standard deviations. Note recovery level is similar to that seen for mCD8-GFP in dendrites (see Fig 7). Drosophila larvae containing the protein trap G00071 [39], Rtnl1-GFP, were used in this experiment.
(TIF)

S5 Fig Ablation of ddaD does not disrupt the proximal axon diffusion barrier of ddaE. Example images are shown for a FRAP assay in which mCD8-GFP was bleached in the proximal axon of a ddaE neuron after laser ablation of ddaD. Note the slow, incomplete recovery in fluorescence, similar to that observed in preparations with intact ddaE neurons (see Fig 7).
(TIF)

S1 Movie After simultaneous bleaching, recovery at the base of the dendrite is much faster than at the base of the axon. mCD8-GFP was expressed in ddaE neurons with the 221-Gal4 driver and fluorescence was bleached at the base of the axon and dendrite simultaneously. A movie including before bleach and a time course after bleaching is presented. The dendrite points up and the axon is below the ddaE cell body.
(MOV)

S1 Table Summary of Ankyrin genes found with BLAST searches of genomes and transcriptome. Genomes and transcriptomes of choanoflagellates and basal metazoans were searched with BLAST using the ZU5-ZU5-UPA region and results are summarized.
(XLSX)

S2 Table Source and amino acid sequences for ankyrins used in phylogenetic analysis. The table contains a list of the accession numbers, full amino acid sequences and aligned sequence used for phylogenetic analysis.
(XLSX)

S3 Table Comparison of Ankyrin long exons to average ORF size in four genomes. The number of ORFs and genome-wide average ORF size are shown and compared to the length of the long exon that encodes the giant Ankyrin.
(XLSX)

S4 Table Summary table of long exons encoding giant isoforms of Ankyrin genes. A list of the *Ankyrin* genes, length of long exon, position, and data on repeats within the long exon is shown.
(XLSX)

S1 Text Long Exon Sequence Analysis. (DOCX)
