# Supplementary figures and images for: Bilaterian Giant Ankyrins Have a Common Evolutionary Origin and Play a Conserved Role in Patterning the Axon Initial Segment
Source: PLoS Genet. 2016 Dec 2;12(12):e1006457. doi: 10.1371/journal.pgen.1006457 (PMC5135030; doi:10.1371/journal.pgen.1006457)

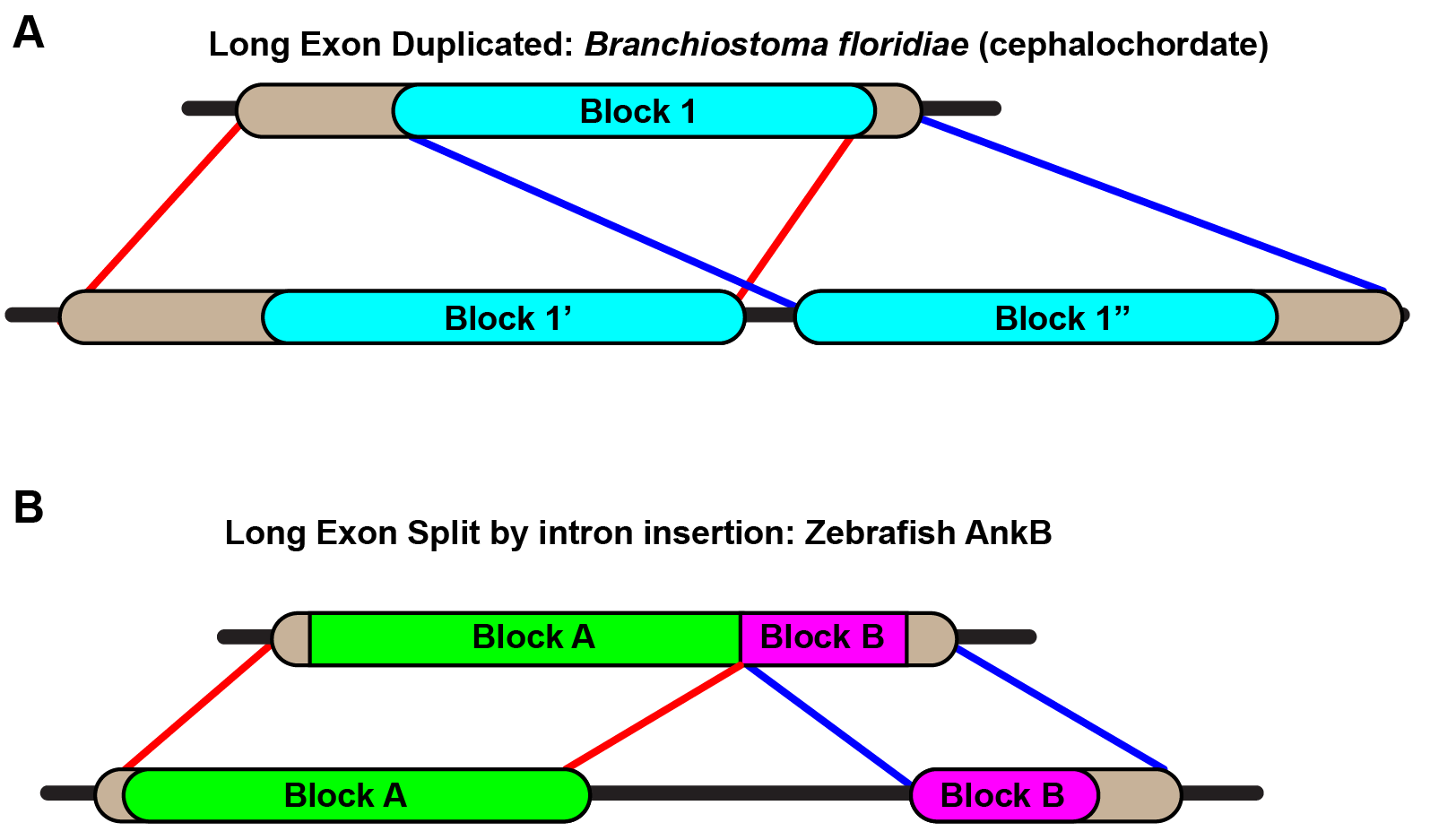

Supplement: S1 Fig — (A) Two long exons in the Branchiostoma floridiae (amphioxus, cephalochordate) share a large block of homology (light blue) and thus appear to arise from a duplication of an ancestral long exon. (B) In Zebrafish AnkB, an intron separates two sequence blocks (green and pink), that are contiguous in AnkB sequences from mouse and human, indicating that the two exons arise from a recent intron insertion into a single ancestral long exon. Similar intron insertions appear to have created multiple long exons in ankyrins from the sea urchin Lytechnicus variegatus and the horseshoe crab Limulus polyphemus (not shown). (TIF) [file pgen.1006457.s001.tif]

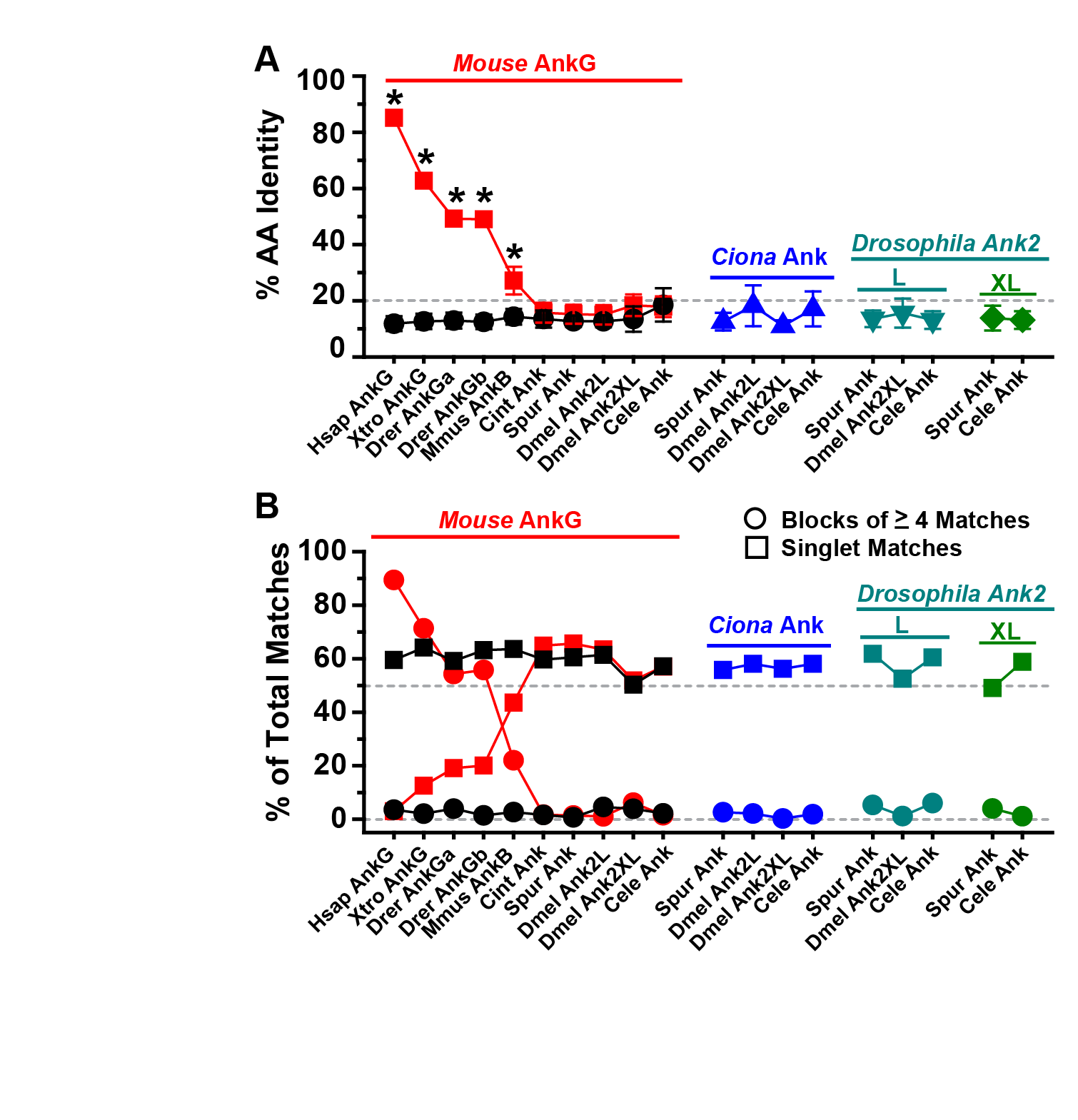

Supplement: S2 Fig — (A) Percent amino acid identity of long exon-encoded polypeptides found in pairwise comparisons. The first long exon polypeptide sequence in each comparison is listed above the colored bar, while the second is listed in the X-axis legend. For mouse AnkG comparisons (red), we also ran controls in which sequence order for the second polypeptide was randomized (black). Data show mean ± S.E.M. of values obtained from 6 different alignment techniques, and asterisks mark values significantly higher than randomized controls (t-test, p < 0.05). The Mouse AnkG long exon shares homology significantly above the randomized control background only with vertebrate AnkG and AnkB orthologs. No significant homology was detected between mouse AnkG and Ciona Ankyrin long exons, which share a common insertion position upstream of the DD. (B) Percent of identities in pairwise alignments generated with the MAFFTWS algorithm occurring as singlets (squares) or in blocks of ≥ 4 consecutive identities (circles). Note that the percent of identities occurring in blocks of ≥ 4 is near zero in randomized controls (black), suggesting that homology blocks of this size are an indicator of true rather than random similarity. In contrast, randomized controls show a high percentage of singlet matches, suggesting that singlet matches in that absence of larger blocks simply represent random background instead of true homology. This homology block analysis agrees with the percent identity analysis in (A) in indicating that the long exon of mouse AnkG only has detectable true homology to long exons from vertebrate AnkGs and AnkBs. All other comparisons show the random control pattern of a high percentage of singlet matches and few if any matches in blocks of ≥ 4. Note both types of comparisons, straight percent identity and homology block analysis, also fail to detect meaningful conservation of long exon sequence between deeply diverging invertebrate species, regardless of exon position. (TIF) [file pgen.1006457.s002.tif]

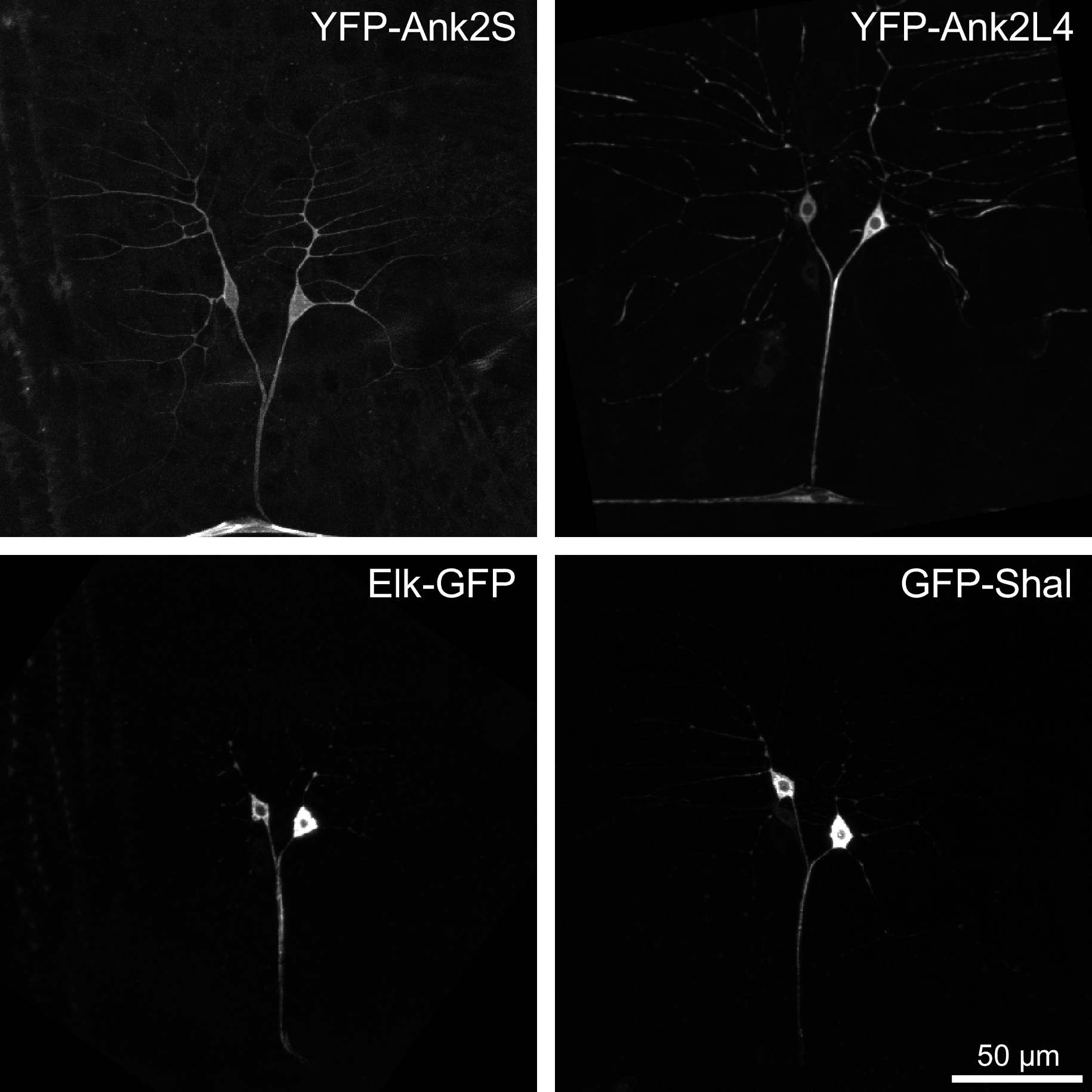

Supplement: S3 Fig — The GFP channel is shown in isolation for the dual color images presented in Fig 6B and 6D to allow a clear view of the distribution of the GFP-fusion protein. (TIF) [file pgen.1006457.s003.tif]

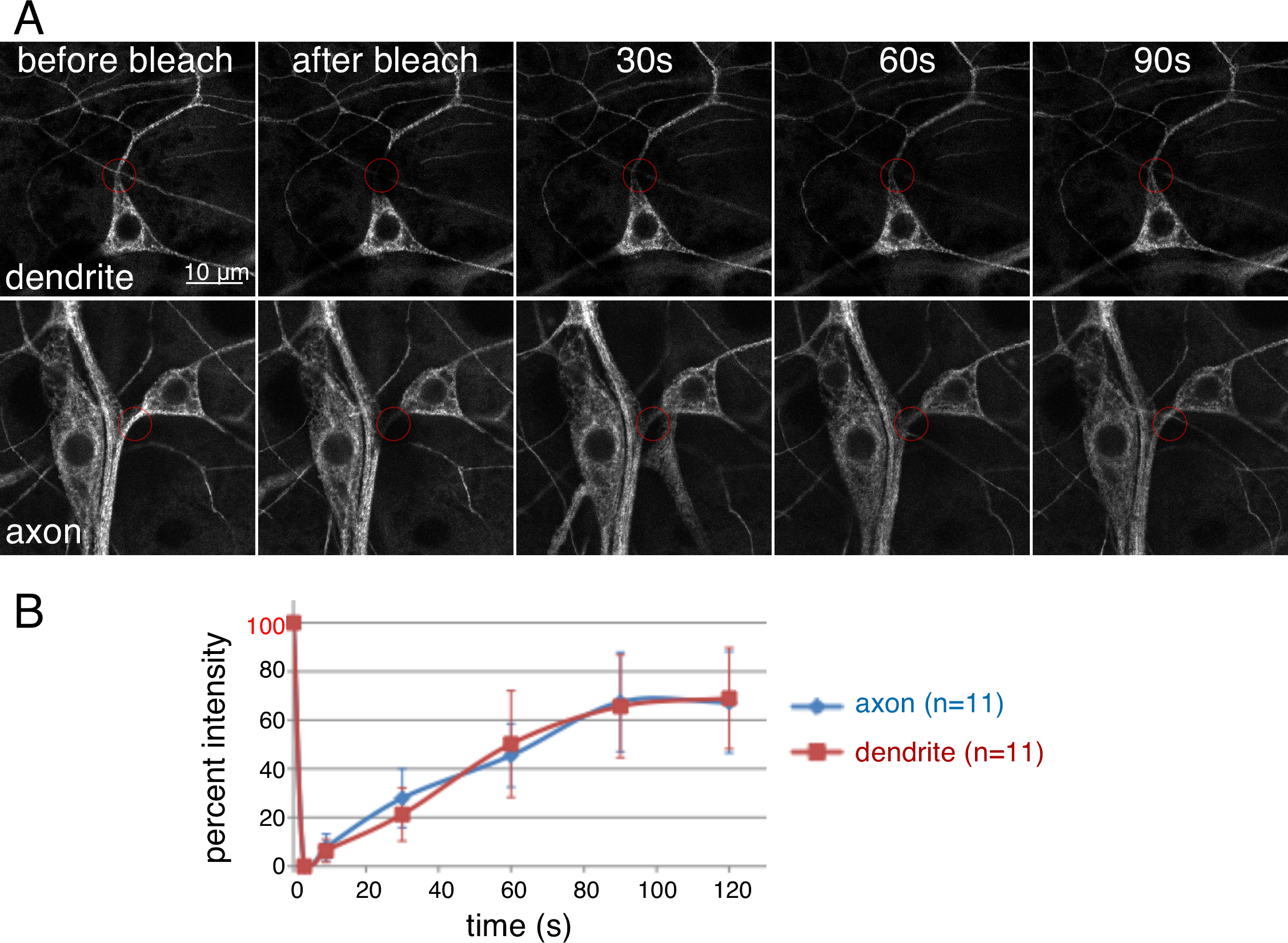

Supplement: S4 Fig — (A) Time series of images showing recovery from photobleaching of Rtnl1-GFP in the proximal dendrite and axon of a ddaE neuron. The bleached region is circled in red. (B) Quantitation of FRAP experiments in 11 animals each for axon and dendrite bleaching is shown. Error bars indicate standard deviations. Note recovery level is similar to that seen for mCD8-GFP in dendrites (see Fig 7). Drosophila larvae containing the protein trap G00071 [39], Rtnl1-GFP, were used in this experiment. (TIF) [file pgen.1006457.s004.tif]

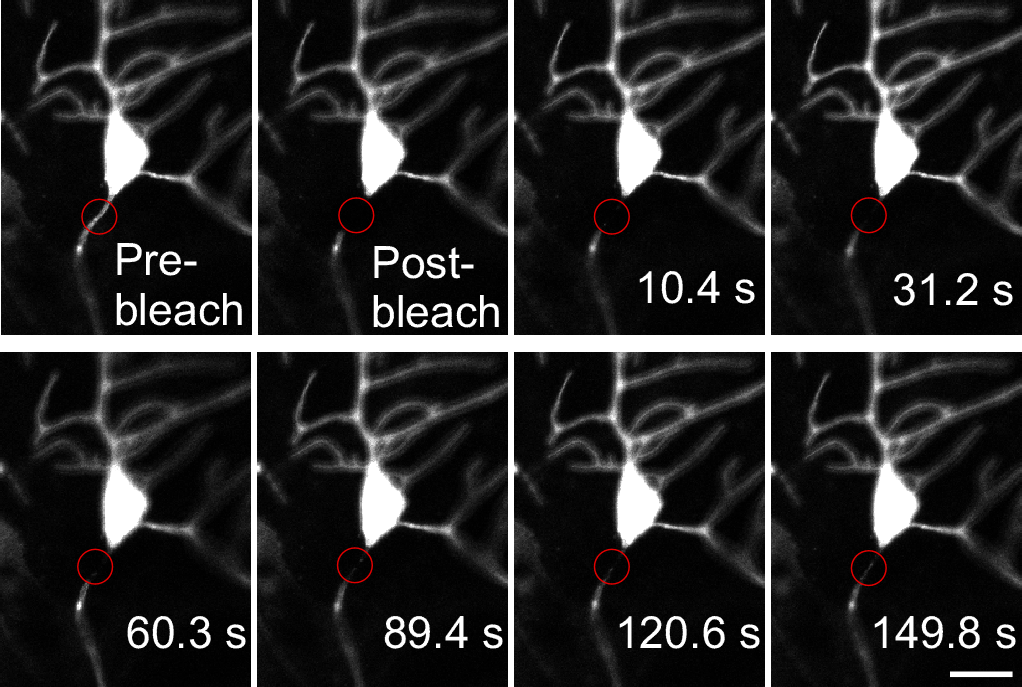

Supplement: S5 Fig — Example images are shown for a FRAP assay in which mCD8-GFP was bleached in the proximal axon of a ddaE neuron after laser ablation of ddaD. Note the slow, incomplete recovery in fluorescence, similar to that observed in preparations with intact ddaE neurons (see Fig 7). (TIF) [file pgen.1006457.s005.tif]
