# Supplementary material for: Bilaterian Giant Ankyrins Have a Common Evolutionary Origin and Play a Conserved Role in Patterning the Axon Initial Segment
Source: PLoS Genet. 2016 Dec 2;12(12):e1006457. doi: 10.1371/journal.pgen.1006457 (PMC5135030; doi:10.1371/journal.pgen.1006457)
Supplement: S1 Text — (DOCX) [file pgen.1006457.s011.docx]

**Long Exon Sequence Analysis**

Multiple sequence alignments of 11 long exon polypeptides (Human, mouse, frog and zebrafish AnkGs; mouse AnkB; *Ciona*, *S. purpuratus*, *C. elegans* Anks; *Drosophila* Ank2 L and XL exons) were generated in Jalview v2.8.2 (3) using CLUSTALWS, MUSCLE, MSAPROBSWS, PROBCONSWS, GLPROBSWS and MAFFTWS under default settings. For each alignment method, pairwise estimates of evolutionary divergence between sequences were calculated in MEGA6 (4) using p-distance. Ambiguous positions were removed for each sequence pair. For each sequence pair, we report the average percentage identity (± SEM) across the 6 alignment algorithms tested. For comparisons derived using MAFFTWS (consistently highest identity of the methods used) we quantified the distribution of aligned residues with an in-house script written in Tcl. We counted the number of identities in various block sizes (singlet to Block_n_) across the alignment length and converted the raw count to frequency in the following manner: Total Number of Identities in Block_n_/Total Number of Identities in Alignment.

For controls, we made pairwise comparisons between wild-type mouse ANKG and randomized long exon sequences with same length and composition as their respective WT counterpart generated using RandSeq (<http://web.expasy.org/randseq/>). We used the same 6 alignment algorithms, and quantified the average pairwise identity (± SEM) and frequency of identity blocks as described above. To assess significance, mouse ANKG vs. WT and mouse AnKG vs. random controls were compared with unpaired two-tailed T-Tests.
